# Diel-Regulated Transcriptional Cascades of Microbial Eukaryotes in the North Pacific Subtropical Gyre

**DOI:** 10.3389/fmicb.2021.682651

**Published:** 2021-09-29

**Authors:** Ryan D. Groussman, Sacha N. Coesel, Bryndan P. Durham, E. Virginia Armbrust

**Affiliations:** ^1^School of Oceanography, University of Washington, Seattle, WA, United States; ^2^Department of Biology, Genetics Institute, University of Florida, Gainesville, FL, United States

**Keywords:** metatranscriptome, phytoplankton, biological oceanography, environmental genomics, protists

## Abstract

Open-ocean surface waters host a diverse community of single-celled eukaryotic plankton (protists) consisting of phototrophs, heterotrophs, and mixotrophs. The productivity and biomass of these organisms oscillate over diel cycles, and yet the underlying transcriptional processes are known for few members of the community. Here, we examined a 4-day diel time series of transcriptional abundance profiles for the protist community (0.2–100 μm in cell size) in the North Pacific Subtropical Gyre near Station ALOHA. *De novo* assembly of poly-A+ selected metatranscriptomes yielded over 30 million contigs with taxonomic and functional annotations assigned to 54 and 25% of translated contigs, respectively. The completeness of the resulting environmental eukaryotic taxonomic bins was assessed, and 48 genera were further evaluated for diel patterns in transcript abundances. These environmental transcriptome bins maintained reproducible temporal partitioning of total gene family abundances, with haptophyte and ochrophyte genera generally showing the greatest diel partitioning of their transcriptomes. The haptophyte *Phaeocystis* demonstrated the highest proportion of transcript diel periodicity, while most other protists had intermediate levels of periodicity regardless of their trophic status. Dinoflagellates, except for the parasitoid genus *Amoebophrya*, exhibit the fewest diel oscillations of transcript abundances. Diel-regulated gene families were enriched in key metabolic pathways; photosynthesis, carbon fixation, and fatty acid biosynthesis gene families had peak times concentrated around dawn, while gene families involved in protein turnover (proteasome and protein processing) are most active during the high intensity daylight hours. TCA cycle, oxidative phosphorylation and fatty acid degradation predominantly peaked near dusk. We identified temporal pathway enrichments unique to certain taxa, including assimilatory sulfate reduction at dawn in dictyophytes and signaling pathways at early evening in haptophytes, pointing to possible taxon-specific channels of carbon and nutrients through the microbial community. These results illustrate the synchrony of transcriptional regulation to the diel cycle and how the protist community of the North Pacific Subtropical Gyre structures their transcriptomes to guide the daily flux of matter and energy through the gyre ecosystem.

## Introduction

The North Pacific Subtropical Gyre (NPSG) is a warm and oligotrophic (nitrogen-limited) ecosystem that hosts a diverse community of phototrophic, heterotrophic, and mixotrophic microbial eukaryotes (protists) spanning over three orders of magnitude in cell sizes. The phylogenetically diverse eukaryotic phytoplankton (phototrophs) represents nearly half of the phytoplankton biomass of the NPSG and are composed primarily of organisms derived through secondary or tertiary endosymbiosis such as dinoflagellates, haptophytes, and ochrophytes (photosynthetic stramenopiles) (Alexander et al., 2015; Hu et al., 2018). Strictly phototrophic members of the NPSG eukaryotic phytoplankton consist of haptophytes including *Emiliania huxleyi* (Hernández et al., 2020) and diatoms such as *Rhizosolenia* and *Hemiaulus* (Villareal et al., 1993), two genera found in symbioses with nitrogen fixing cyanobacteria that bloom during sporadic injections of nutrients into the surface waters. The strictly heterotrophic (zooplankton) component of the protist community is dominated by the Alveolata supergroup (including dinoflagellates), as well as stramenopiles and Rhizaria (Rii, 2016; Hu et al., 2018). Many eukaryotic lineages within the NPSG have mixotrophic life strategies, adjusting their relative balance of photosynthesis and phagotrophy to changing light and nutrient conditions (Mitra et al., 2016; Lambert et al., 2021). In addition, mixotrophs can be distinguished between constitutive (vertical inheritance of plastids) and non-constitutive (kleptoplastic, or acquisition of plastids from prey) (Mitra et al., 2016). Members of haptophyte, ochrophyte, and dinoflagellate lineages are constitutive mixotrophs (Faure et al., 2019), with evidence that they can graze on picocyanobacteria in the NPSG (Frias-Lopez et al., 2009). In the well-lit and low nutrient conditions of the NPSG, mixotrophy may be advantageous (Rothhaupt, 1996), and the gene family abundance profiles of many environmental protist species suggest widespread mixotrophy (Lambert et al., 2021). Some ciliates, such as *Strombidium*, are non-constitutive mixotrophs that retain the plastid of their consumed prey (Stoecker et al., 2009; Faure et al., 2019).

The daily cycles of light and darkness synchronize cell growth and division across the diverse members of the microbial communities within the sunlit waters of the NPSG. For example, the phototrophic cyanobacteria *Prochlorococcus* displays reproducible increases in mean cell diameter over the day as cells fix carbon into biomass, followed by decreases during the night as cells undergo cell division (Vaulot and Marie, 1999; Ribalet et al., 2015). From day to day there is little variation in the total cellular abundance of *Prochlorococcus*, indicating that growth rates roughly equal loss rates in an ecological system finely tuned to the daily solar cycle (Ribalet et al., 2015). The tight coupling of growth and division to the light/dark cycle is also clear within small eukaryotic protists (<10 μm size fraction), although the different species underlying the observed changes in biomass cannot be distinguished based on the optical measures (Freitas et al., 2020). Heterotrophic bacteria also display oscillating waves of species-specific transcriptional patterns over the diel cycle (Ottesen et al., 2014), potentially due to the daily cycle of phytoplankton production of organic matter.

Recent ‘omics analyses have enhanced our understanding of the metabolic cascades resulting from synchrony to the daily light cycle in the NPSG. For example, transcript abundances in the haptophyte phototroph *E. huxleyi* underwent daily oscillations, with transcripts associated with carbon fixation and lipid synthesis proteins reaching a peak in abundance near dawn and at mid-day or dusk for those genes encoding respiration and lipid degradation, suggesting a cycle of energy-store biosynthesis and consumption (Hernández et al., 2020). A similar pattern was observed in the more nutrient-rich waters of the California Current upwelling, with oscillations in the abundance of diatom and green algae photosynthesis-related transcripts during the morning and cell division-related transcripts during the night (Kolody et al., 2019). Organisms in the NPSG are equipped with a diversified repertoire of photoreceptors (Coesel et al., 2021), which may allow them to sense and regulate their daily responses to changes in light conditions. Responses to the light cycle include the biosynthesis of energy-rich triacylglycerols during the day and their consumption during the night (Becker et al., 2018) and the replenishment of pigments during the night to compensate for photodegradation during the day (Becker et al., 2020). Moreover, a diel structuring of cross-kingdom interactions was demonstrated by the species-specific exchange of phytoplankton-produced organic sulfonated compounds, produced primarily by haptophytes and diatoms and consumed by heterotrophic bacteria (Durham et al., 2019).

Here, we examined diel transcriptional patterns across the microbial eukaryotes (protists) (0.2–100 μm in size) of the NPSG to evaluate how metabolic pathways may be synchronized across the microbial eukaryote community and the varied trophic states that comprise it. We hypothesized that eukaryotic gene expression would be strongly patterned by evolutionary lineage and trophic level, with phototrophs and mixotrophs expected to demonstrate the highest degree of diel periodicity in their transcriptome and, by inference, their metabolism. Analyses centered on the direct annotation of metatranscriptome-assembled contigs allowed us to investigate large-scale transcriptional patterns in abundant eukaryotic taxa and to identify functional metabolic processes operating on diel cycles.

## Materials and Methods

### Cruise and Sample Collection

Duplicate samples for eukaryotic metatranscriptomes were collected from 15-m depth every 4 h (06:00, 10:00, 14:00, 18:00, 22:00, and 02:00 HST) over the course of 4 days on the R/V *Kilo Moana* cruise KM1513 (July and August 2015) approximately 100 km NE of Station ALOHA in the North Pacific Subtropical Gyre (see Wilson et al., 2017 for additional cruise details). For each of the 48 samples, 7 L of seawater was pre-filtered through a 100-μm nylon mesh and collected onto a 142-mm 0.2-μm polycarbonate filter using a peristaltic pump. Filters were immediately flash-frozen in liquid nitrogen and subsequently stored at −80°C until further processing. Filters were extracted using the ToTALLY RNA Kit (Invitrogen, Carlsbad, CA, United States) with some modifications. Briefly, frozen filters were added to 50-ml falcon tubes containing 5 ml of denaturation solution and extraction beads (125-μl 100-μm zirconia beads, 125-μl 500-μm zirconia beads, and 250-μl 425–600-μm silica glass beads). A set of 14 internal mRNA standards was added to the extraction buffer for each sample to generate quantitative transcript inventories; these standards were synthesized as previously described (Satinsky et al., 2013), with the exception that eight standards were synthesized with poly(A) tails to mimic eukaryotic mRNAs. Total extracted RNA was treated with DNase I (Ambion, Grand Island, NY, United States) and purified with DNase inactivation reagent (Ambion). Eukaryotic mRNAs were poly(A)-selected, sheared to ∼225-bp fragments, and used to construct TruSeq cDNA libraries according to the Illumina TruSeq^®^ RNA Sample Preparation v2 Guide for paired-end (2 × 150 bp) sequencing using the Illumina NextSeq 500 sequencing platform.

### DNA Sequence Quality Control and *de novo* Assembly

Raw Illumina sequence reads were quality controlled with trimmomatic v0.36 (Bolger et al., 2014, parameters: *MAXINFO:135:0.5, LEADING:3, TRAILING:3, MINLEN:60*, and *AVGQUAL:20*). A total of 2,426,923,906 merged paired-end sequences were generated with a median length of ∼240 bp. Sequences were pooled for each of the 24 sampling times, and the 24 pooled samples were assembled using the Trinity *de novo* transcriptome assembler v2.3.2 (Grabherr et al., 2011, parameters: –normalize_reads –min_kmer_cov 2 –min_contig_length 300) on the Pittsburgh Supercomputing Center’s Bridges Large Memory system. Trinity assembly yielded 52,489,585 total contigs from all 24 assemblies.

### Quality Control of Assemblies, Translation, and Longest Frame Selection

The raw assemblies were quality controlled with Transrate v1.0.3 (Smith-Unna et al., 2016) to check contigs for chimeras, structural errors, and base errors, using their paired-end assembly method (parameters: –assembly $sample.fasta –left $left_reads –right $right_reads). A total of 31,284,431 contigs (59.6% of the raw pool) passed the optimized assembly score threshold and were retained for further analysis. The quality-controlled contigs were translated in six frames with transeq vEMBOSS:6.6.0.059 (Rice et al., 2000) using the Standard Genetic Code. The longest (or multiple frames if of equal lengths) open reading frame from each contig was retained for downstream analyses. A total of 32,536,410 translated frames that passed these criteria were retained.

### Clustering

The 24 peptide assemblies were merged and clustered at the 99% identity threshold level with linclust within the MMseqs2 package (version 31e25cb081a874f225d443eec307a6254f06a291, Steinegger and Söding, 2018, –min-seq-id 0.99). A total of 30,015,008 peptide sequences (92% of input sequences) were retained as cluster representatives and used for further analysis.

### Annotation

Following clustering at 99% identity, representative contigs were annotated for taxonomy and function. Contigs were annotated against the curated MarineRefII reference database^1^ of 641 marine eukaryotes and prokaryotes, including the MMETSP transcriptomes (Keeling et al., 2014), and supplemented with available marine animal, fungal, choanoflagellate, and viral sequences (Coesel et al., 2021). Assembled contigs were aligned to the reference database using DIAMOND v 0.9.18 (Buchfink et al., 2014, parameters: –sensitive -b 65 -c 1 -e 1e-5 –top 10 -f 100). The Lowest Common Ancestor (LCA) was estimated using the LCA algorithm in DIAMOND in conjunction with NCBI taxonomy. The frame with the lowest (best) *e*-value was retained if multiple frames of a contig received annotations. A total of 15,302,768 contigs were assigned an NCBI tax_id (51.0%). Contigs assigned to the same NCBI taxon or daughter nodes were defined as members of the same environmental taxon “bin.” To determine the putative function of each contig, we used hmmsearch, from HMMER 3.1b2 (Eddy, 2011, parameters: -T 30 –incT 30), to assign KEGG Orthology IDs (KOs) to contigs using 22,247 hmm profiles from KOfam ver. 2019-03-20 (Aramaki et al., 2020). The profile with the highest bitscore was retained for those contigs that mapped to more than one KOfam profile. If multiple frames of a contig received annotations, the frame with the highest annotation bitscore was retained. A total of 7,707,191 contigs were assigned an KEGG KO (25.7%).

### Quantification and Normalization of Abundance

The clustered contig representatives were quantified by alignment of their nucleotide sequences against the paired-end reads using kallisto v0.43.1 (Bray et al., 2016, parameters: quant –rf-stranded -b 40). We normalized contigs abundance to fragments per kilobase of transcript per million total reads (FPKM), using total read values mapped to each taxonomic bin, rather than the total library size, as the denominator, “M.” FPKM values for contigs with the same taxonomy and functional annotation combinations (NCBI tax_id and KEGG KO) were summed. Environmental taxa bins were integrated down taxonomic levels, summing abundance values from lower-ranking nodes in the NCBI taxonomy.

### NMDS Ordination

Non-metric multidimensional scaling (NMDS) ordination was used to reduce taxonomic, temporal, functional, and abundance information into three-dimensional space. The input was a matrix of observations consisting of each of the 48 genus-level taxa for each of the 24 time points (1,152 total observations). The features for each observation were the *in silico* normalized counts for the 6,925 KOfams present in >5% of the observations. The features within observations were normalized such that the row sums equal 1. The metaMDS function in the R package “vegan” version 2.5-5 (Oksanen et al., 2019, parameters: *k* = 3, trymax = 100) was used to compute the Bray–Curtis distance matrix and find a solution between runs. A solution for this ordination with two dimensions was not achievable. The three-dimensional NMDS ordination results were visualized with the R package “plotly” (Sievert, 2018). NMDS ordinations were also individually generated for the 48 genera. The individual ordinations were resolved in two dimensions (parameters: *k* = 2). Mean stress of 48 NMDS = 0.127 ± 0.028 stdev.

### Determining Significant Diel Periodicity

Significant periodicity of gene family abundances for each genus was determined with the Rhythmicity Analysis Incorporating Non-parametric Methods (RAIN) package implemented in R (Thaben and Westermark, 2014). The *p*-values from RAIN analyses were ranked and corrected at an FDR < 0.05 using the Benjamini–Hochberg false-discovery rate method (Benjamini and Hochberg, 1995), as implemented in Ottesen et al. (2014).

### Enrichment of Significantly Diel Gene Families in KEGG Pathways

The KEGG pathways and their associated knums (gene family identifiers representing a KOfam) were parsed using the R package KEGGREST (Tenenbaum, 2016) to access the KEGG database. Only pathways with associated knums were used. A contingency matrix for each genus-pathway-time combination (a total of 124,704 combinations of contingency matrices for genus-level analysis, and 106,518 for order-level analysis) was constructed for the test and populated with the appropriate counts: “knum in pathway and is diel,” “knum is in pathway and is not diel,” “knum is not in pathway and is diel,” and “knum is not in pathway and is not diel.” The “diel” status of each knum was determined from the RAIN results, above. We removed contingency matrices with no identified knums in the pathway, reducing our number of tests to 108,288 for the genus-level analysis and 93,114 for the order-level analysis. We used Fisher’s Exact Test on these matrices to determine enrichment, combined with a Benjamini–Hochberg multiple comparison correction and false discovery rate of less than 5% (genera-level adjusted maximum *p*-value of <3.412673e-05, order-level adjusted maximum p-value of 5.792821e-05) (Benjamini and Hochberg, 1995), both executed within R.

## Results

### Taxonomic and Functional Composition of Environmental Transcriptome Bins

We examined the transcriptional profiles of eukaryotic microbes (protists) over the diel cycle by collecting size-fractionated (0.2–100 μm) RNA samples every 4 h (at 06:00, 10:00, 14:00, 18:00, 22:00, and 02:00 HST) over 4 consecutive days in the oligotrophic North Pacific Subtropical Gyre (NPSG), ∼100 km NE of Station ALOHA. Local sunrise was at ∼06:00 and sunset was at ∼18:00 HST. Surface illumination intensities reached over 2,000 μmol m^–2^ s^–1^ between 10:00 and 14:00 HST over the 4-day sampling period, during which the picoeukaryotic phytoplankton grew and divided on an oscillating daily basis, as estimated from continuous flow cytometry measurements (Coesel et al., 2021). Water column properties during the cruise confirm a warm (26.6–27.04°C with some diel variability) and nitrogen-deplete environment (nitrate + nitrite 8 ± 4 nmol L^–1^) (Wilson et al., 2017). Casts were taken at 15 m depth, with the research vessel (KM1513) tracking a Lagrangian drogue placed at 15 m to allow repeat sampling of the planktonic community from the same water mass. Illumina deep sequencing of libraries created from poly-A+ selected RNA yielded a total of ∼2.4 billion transcript fragments (merged paired-end reads) with an average merged length of ∼240 bp (Table 1). *De novo* assembly of the metatranscriptome sequences generated about 52 million nucleotide contigs. Subsequent quality control, translation, frame selection, and clustering at 99% amino acid identity (Table 1) yielded 30 million amino acid sequences (hereafter referred to as “contigs”), with an N50 of 423 base pairs (141 amino acid residues, Supplementary Figure 1 and Table 1).

**TABLE 1:** Sequence data metrics.

Sequence data	Count	Type
Merged read pairs	2,426,923,906	Nt
Raw contigs	52,489,585	Nt
QCed contigs (transrate)	31,284,431	Nt
Translated, clustered contigs	30,015,008	Aa
Contigs w/any taxonomy	16,061,543	Aa
Contigs w/any KEGG function	7,707,191	Aa
Genus-level taxonomy	5,181,384	Aa
Genus-level w/KEGG function	1,390,232	Aa

*Volume of sequence data by short reads, assembled contigs, and annotated contigs. Type refers to nucleotide (“nt”) or translated amino acid (“aa”) sequence. Genus-level taxonomy and functional counts include contigs assigned to nested daughter taxonomies.*

**FIGURE 1:**
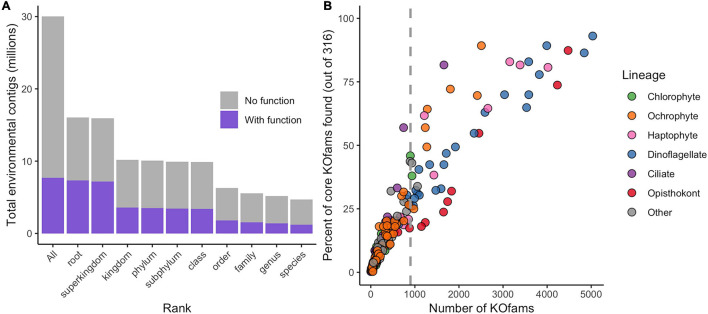
Annotation of metatranscriptome assemblies by taxonomy, function, and abundance. **(A)** Cumulative number of contigs placed at each rank or lower. “All” includes taxonomically unassigned contigs. **(B)** Completeness of genus-level taxonomic bins based on total number of KOfams and percent of the 316 “core KOfams.” Circles represent genus-level taxonomic bins, colored by lineage as in legend. Dashed line represents cutoff criteria of 900 detected KOs for subsequent analyses.

The translated contigs were annotated in two ways. First, taxonomic identity and rank within the NCBI taxonomic framework were determined by mapping contigs against a reference database of 18.5 million predicted protein sequences from 554 marine eukaryotes, bacteria, archaea, and viruses (Coesel et al., 2021) and estimating the Lowest Common Ancestor (LCA) of matches. Placement to any taxonomic level was possible for 16 million contigs (51% of total; Figure 1A, Table 1, and Supplementary Figure 2); the remaining 49% received no taxonomic annotation. Bacteria or Archaea were assigned to 55,099 and 740 contigs, respectively; these contigs were not considered further in this study. Second, potential function was assigned by mapping the contigs against the Kyoto Encyclopedia of Gene and Genomes (KEGG) database of orthogenes (KOfams) using HMM profiles (Aramaki et al., 2020). A total of 7.7 million contigs (25.7%) were assigned to a putative KOfam and the associated KO term, with 13,765 total KOfams identified among all contigs (Figure 1A and Table 1). Approximately 5.2 million contigs were annotated to a genus rank or lower (Figure 1A and Table 1), with a subset of 1.4 million contigs that also received a putative KEGG function. This set of 1.4 million contigs with putative genus-level taxonomy and function were grouped based on their assigned genus-level taxonomy to create environmental taxonomic bins and used for subsequent analyses. Two hundred thirty-one environmental eukaryotic genera bins were represented by at least one functional KOfam assignment (Figure 1B). A low proportion of reads aligned to reference sequences of metazoans (opisthokonts) larger in size than 100 μm, potentially reflecting varied biological sources including sloughed cells, gametes, and other life cycle stages.

We developed a metric of transcriptome completeness to identify a subset of well-represented environmental genera from this initial set of taxonomic bins for further analyses. We first estimated a minimum number of KOfams expected within marine eukaryotes by *de novo* mapping the proteomes of our 366 reference marine eukaryotes against 22,247 KOfam hmm profiles (ver. 2019-03-20; Aramaki et al., 2020). Three hundred fifty-five of the reference protists (over 96% of eukaryotic reference taxa) each contained 900 or more KOfams; only parasitic protists with a reduced gene content possessed significantly fewer KOfams (Supplementary Figure 3 and Supplementary Data Sheet 1). A set of 316 KOfams were defined as “core KOfams” due to their presence in ∼95% of reference marine eukaryotes (Supplementary Data Sheet 2). The completeness of a given genus-level environmental taxonomic bin was estimated based on the percentage of core KOfams identified in the environmental bins (Figure 1B and Supplementary Figure 3). We constrained further analysis to 48 environmental genera bins that each had >900 detected KOfams. These 48 genera were grouped by eight higher-level lineages (Supplementary Data Sheet 3): dinoflagellates (*n* = 23), opisthokonts (*n* = 8), ochrophytes (photosynthetic stramenopiles, *n* = 7), haptophytes (*n* = 6), ciliates (*n* = 1), chlorophytes (*n* = 1), and two “other” lineages of kinetoplastids and oomycetes. These 48 genera corresponded to genera previously detected in the NPSG through metabarcoding (Hu et al., 2018) and collectively had an average of 54% (170 of 316) core KOfams positively identified (Supplementary Data Sheet 3). The highest proportion of core KOfams were detected in the taxonomic bin most closely related to the dinoflagellate *Karlodinium* (93%). Confidence values (*e*-value) for taxonomic assignments were assessed for all 48 genera, with the majority of contigs assigned to each bin receiving the highest-possible confidence value of 0 (Supplementary Figure 4). Some genera displayed a shallower distribution of *e*-value placements, reflecting a more likely entrainment of distantly related taxa to genus representatives. This is most noticeable in multicellular metazoans with single genus reference representatives (e.g., *Octopus*, *Salmo*, and *Orcinus*) and some dinoflagellates (*Amphidinium*, *Kryptoperidinium*, and *Lingulodinium*).

### Dimensionality Reduction of Environmental Transcriptome Bins

**FIGURE 2:**
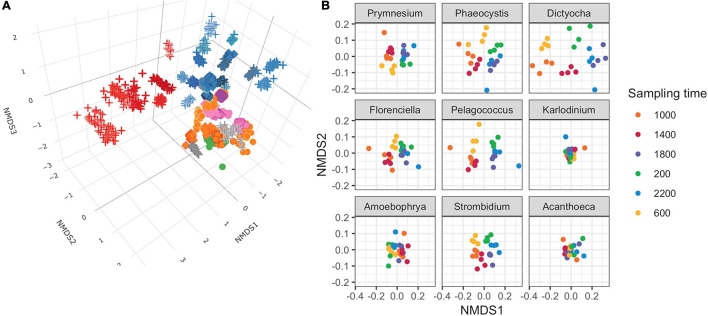
Non-metric multidimensional scaling (NMDS) ordinations of transcript abundances from 48 genus-level environmental bins. **(A)** Three-dimensional ordination for 48 genus-level taxonomic bins meeting completeness criteria. Stress = 0.160. Each point is a discrete time point for each genus based on the mean transcript abundances for each KOfam from duplicate samples. Shapes indicate assumed trophic level: circle, phototroph; diamond, mixotroph; cross, heterotroph. Inner color corresponds to Lineage in legend from Figure 1B; outer color is unique to genera within a lineage. **(B)** NMDS ordination performed independently on gene families belonging to nine representative protist genera. Mean stress of nine independent NMDS = 0.114 ± 0.025 stdev.

Non-metric multidimensional scaling (NMDS) of Bray–Curtis dissimilarity was used to compare the similarity of transcript abundances for thousands of gene families across the 48 genus-level environmental bins over the 24 time points (Figure 2A). The input matrix consisted of each of the 48 genus-level environmental bins for each of the 24 time points (1,152 total observations) and the row-normalized number of transcripts associated with KOfams present in >5% of observations (6,925 total features). Several patterns emerged from the resulting 3D NMDS ordination. First, the observations clustered together according to genus designation rather than time point, indicating that each taxonomic bin displayed a relatively distinct transcriptional fingerprint irrespective of the time of sampling. Second, phylogenetically related genera clustered near one another. One notable exception to this pattern was the environmental bin corresponding to the dinoflagellate genus *Amoebophrya*, a highly-derived genus of dinoflagellates with a parasitoid life cycle (Guillou et al., 2008). Third, both the dinoflagellate genera and opisthokont genera (seven metazoa and one choanoflagellate) clustered apart from other protists. Among the remaining protists, known phototrophic eukaryotes (diatoms, chlorophytes, and some haptophytes), heterotrophs, and potential mixotrophs formed distinct clusters, with the positioning of putative mixotrophs between the heterotroph and autotroph clusters reflecting their mixed metabolism.

We conducted independent NMDS ordinations on each of the 48 environmental genera bins to evaluate whether temporal partitioning of transcript abundances resolved within each environmental bin. Individual NMDS ordinations for the 48 genera were generated (mean stress = 0.114 ± 0.025 stdev) (Supplementary Figure 5). We highlighted a select subset of representative genera as representatives of the NPSG protist community in Figure 2B. We focused on nine representative environmental protist genera, based on their high sequence coverage, a high proportion of total and core KOfams, and their representation of different trophic states and evolutionary lineages (Table 2 and Supplementary Data Sheet 3). Haptophytes with contrasting trophic modes were represented by the genus *Prymnesium* (order Prymnesiales), which contains known mixotrophic species (Faure et al., 2019) and the genus *Phaeocystis* (order Phaeocystales), which is considered strictly photosynthetic and can exist as a free-living flagellate or in a colonial form (Rousseau et al., 2007). Ochrophytes were represented by the silicoflagellate genus *Dictyocha* (order Dictyochales), the genus *Florenciella* (order Florenciellales), and the pico-eukaryotic (<2 μm cell size diameter) genus *Pelagococcus* (order Pelagomonadales). Both *Dictyocha* and *Florenciella* include known mixotrophic species (Quéguiner, 2016; Li et al., 2021), whereas *Pelagococcus* is thought to be strictly photosynthetic (Lewin et al., 1977). Dinoflagellates were represented by the genus *Karlodinium* (order Gymnodiniales), which contains mixotrophic species, similar to many of the dinoflagellate genera observed in this study (Faure et al., 2019). Isotopically labeled grazing experiments on *Prochlorococcus* and *Synechococcus* in the NPSG identified prymnesiophytes (Prymnesiophyceae), dictyophytes (Dictyochophyceae), and dinoflagellates (Dinoflagellata) as grazers of picocyanobacteria (Frias-Lopez et al., 2009). We also highlighted *Amoebophrya* (order Syndiniales), a parasitic dinoflagellate that infects eukaryotic host cells, because of its distinct lifestyle and because of its notable abundance in this environment as evidenced by the rRNA/rDNA libraries from the NPSG (Hu et al., 2018). The ciliate genus *Strombidium* (order Oligotrichida) can live heterotrophically as well as by non-constitutive mixotrophy through retention of plastids from engulfed prey (Stoecker et al., 2009). The choanoflagellate genus *Acanthoeca* (order Acanthoecida) is thought to be an obligate heterotroph.

**TABLE 2:** General features of nine representative genera.

Genus	Order	Class	Putative trophic mode	References
*Prymnesium*	Prymnesiales	Haptophyta	Mixotrophic	Faure et al., 2019
*Phaeocystis*	Phaeocystales	Haptophyta	Phototrophic	Rousseau et al., 2007
*Florenciella*	Florenciellales	Ochrophyta	Mixotrophic	Quéguiner, 2016; Li et al., 2021
*Dictyocha*	Dictyochales	Ochrophyta	Photo- or mixotrophic	Quéguiner, 2016
*Pelagococcus*	Pelagomonadales	Ochrophyta	Phototrophic	Lewin et al., 1977
*Karlodinium*	Gymnodiniales	Dinophyceae	Mixotrophic	Faure et al., 2019
*Amoebophrya*	Syndiniales	Dinophyceae	Parasitic	Guillou et al., 2008
*Acanthoeca*	Acanthoecida	Choanoflagellata	Heterotrophic	
*Strombidium*	Oligotrichida	Spirotrichea	Hetero- or mixotrophic	Faure et al., 2019

*Taxonomic levels are from the NCBI taxonomic framework.*

The temporal partitioning of transcript abundances in the individual NMDS ordinations (Figure 2B and Supplementary Figure 5) showed that sampling time was an important driver for the transcript ordination for a majority of genera, with samples clustered by collection time and organized in a clock-like fashion. Whereas most genera showed some form of diel partitioning, the transcriptional profiles of dinoflagellates such as *Karlodinium* and *Amoebophrya*, the choanoflagellate *Acanthoeca* (Figure 2B and Supplementary Figure 5), and the heterotrophic protists kinetoplastid *Neobodo* and the stramenopile *Phytophthora* (Supplementary Figure 5) were not distinguished by sampling time, indicating that not all organisms entrain their transcriptional activity to the diel cycle.

### Diel Signatures of Environmental Transcriptome Bins

**FIGURE 3:**
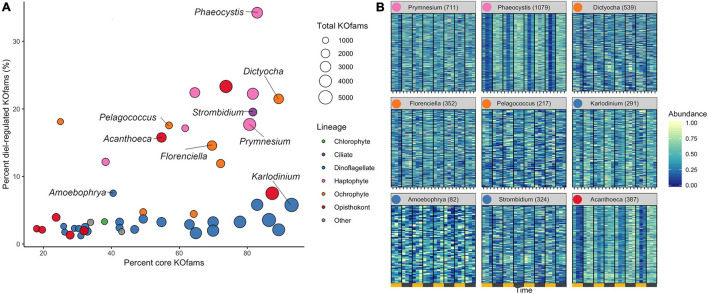
Diel periodicity in environmental genus bins. **(A)** Estimated completeness and diel periodicity of gene family transcript abundance in 48 genera from Figure 1B. Each circle represents one environmental genus bin. The area is proportional to the total number of KOfams identified in the genus. The nine representative genera (Figure 2B) are labeled. **(B)** Normalized abundance heat map of significantly periodic gene families from nine representative protist genera. Yellow and gray bars denote light (06:00, 10:00, and 14:00 HST) and dark (18:00, 22:00, and 02:00 HST) periods, respectively. Numbers in parentheses indicate the number of significantly periodic gene families (FDR < 0.05). Each row corresponds to a gene family, ordered by hierarchical clustering of abundance patterns. Color corresponds to row-normalized abundance values for each gene family. Colored dot by genus name corresponds to Lineage from **(A)**. The order and presence of gene families is not maintained between facets.

We determined the proportion of oscillating transcripts within each of the 48 environmental genera. The diel periodicity of transcript abundances across the 48 environmental genera was tested for a combined total of 103,904 KOfam gene families (RAIN analyses, maximum *p*-value of 0.0044, FDR < 0.05) (Figure 3A and Supplementary Data Sheet 4). Statistically significant diel periodicity in transcript abundance was detected for 9,153 gene families, with peaks in abundance assigned to one of the six sampling times: 06:00, 10:00, 14:00, 18:00, 22:00, and 02:00 HST (Supplementary Figure 6 and Supplementary Data Sheet 4). Haptophytes displayed the highest proportions of diel-oscillating transcript abundances for different gene families (Figure 3A and Supplementary Data Sheet 4). About 34% of the *Phaeocystis* and about 18% of *Prymnesium* gene families underwent significant oscillations in transcript abundance. Most ochrophyte environmental genera displayed diel periodicity in transcript abundance in at least 15% of their gene families, with the highest proportion (21.5%) observed in the environmental silicoflagellate *Dictyocha* (Figure 3A and Supplementary Data Sheet 3). The dinoflagellates displayed a low proportion (average of less than 3%) of gene families with diel transcript abundance patterns, as did the purely heterotrophic opisthokonts (Figure 3A). The heterotrophic environmental stramenopile *Phytophthora* displayed a comparably low (3.2%) proportion of diel gene families. Three other heterotrophic organisms stood out in this analysis. The environmental genera of *Lepeophtheirus* (copepod), *Acanthoeca* (choanoflagellate), and *Strombidium* (ciliate) each displayed relatively high proportions of diel oscillations in transcript abundance across gene families (23, 16, and 20%, respectively), comparable to the haptophytes and non-diatom ochrophytes (Figure 3A and Supplementary Data Sheet 3). Thus, the extent of diel periodicity was not directly correlated with trophic mode and appeared instead to be taxa specific.

The transcription profiles of diel oscillating gene families (KOfams) were evaluated for the nine representative genera (Figures 3A,B) along with the remaining 41 environmental taxa (Supplementary Figures 6, 7). The representative environmental haptophyte *Phaeocystis* showed a transcriptional minimum at 10:00 (Figure 3B), coinciding with environmental light intensities reaching 2,000 μmol/m/s at the sea surface. Similar mid-morning minima are seen in *Acanthoeca* (Figure 3B) and other opisthokonts (Supplementary Figures 6, 7). Transcriptional patterns of the ochrophytes *Florenciella*, *Dictyocha*, and *Pelagococcus*, and the ciliate environmental genus *Strombidium* are more equally distributed across the 24-h diel cycle (Figure 3B). The dinoflagellate *Karlodinium* and most (21 of 23) other dinoflagellate environmental genera (Supplementary Figures 6, 7) displayed relatively minimal distinction of transcript abundances by time (Figure 3B), whereas the parasitic dinoflagellate *Amoebophrya* (Figure 3B) maintained a relatively high proportion (7.5%) of diel oscillating transcript abundances (Supplementary Data Sheet 3). We retrieved sufficient sequences with similarity to representative multicellular metazoan (animal) genera in our reference database (*Capitella*, *Orcinus*, *Octopus*, *Salmo*, *Lepeophtheirus*, *Nematostella*, and *Oikopleura*) to pass our cut-off criteria. Each of these environmental “genera” displayed some temporal partitioning (Figure 3A and Supplementary Figures, 5–7), particularly those identified as *Capitella* (annelid) and *Octopus* (mollusk). We constrained further analysis to protistan genera, recognizing that many metazoan taxa undergo substantial vertical migration over diurnal cycles. Diel vertical migration has also been observed in some species of motile phytoplankton (Shikata et al., 2015), but we assume that the relatively minor swimming speed of migrating protists is not a significant factor in the well-mixed surface layer of the NPSG. Overall, haptophyte and ochrophyte genera tended to display the highest proportions of diel oscillating gene families, regardless of whether the examined genera were primarily mixotrophic or photoautotrophic.

### KEGG Pathways With Diel-Oscillating Transcript Levels

**FIGURE 4:**
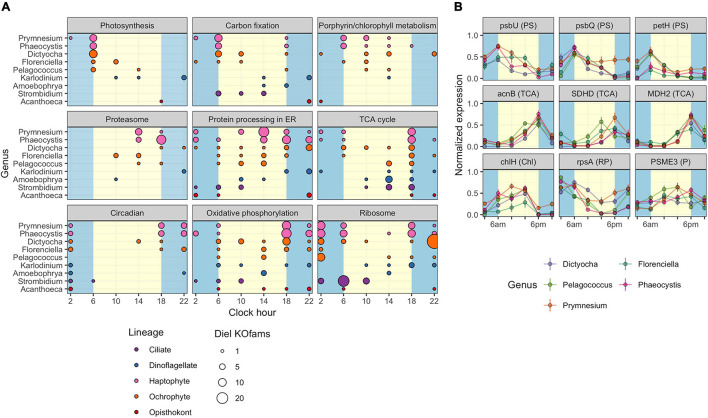
Diel periodicity of metabolic pathways and gene families in select genera. **(A)** Peak times for gene families in select KEGG pathways for nine genera. Circles indicate peak timing for KOfams with significant periodicity (FDR < 0.05), with circle size scaled by the number of KOfams peaking at a given time. Blue and yellow shading denote night and day periods, respectively. **(B)** Temporal abundance of nine gene families from pathways in **(A)**. Haptophytes and ochrophytes from **(A)** are shown. Transcript abundances are min–max normalized. Lines connect the mean expression level across all identical sampling times (two replicates and 4 days). Vertical bars are standard error (*n* = 8). Blue and yellow shading denote night and day periods, respectively. Full protein names are psbU, photosystem II PsbU protein; psbQ, photosystem II oxygen-evolving enhancer protein 3; petH, ferredoxin-NADP+ reductase; acnB, aconitate hydratase 2; SDHD, succinate dehydrogenase membrane anchor subunit; MDH2, malate dehydrogenase; chlH, magnesium chelatase subunit H; rpsA, small subunit ribosomal protein S1; PSME3, proteasome activator subunit 3. Letters in parentheses next to the protein symbol indicate KEGG pathways: PS, Photosynthesis metabolism; TCA, TCA Cycle; N, Chl, Porphyrin and chlorophyll metabolism; RP, Ribosomal protein; P, Proteasome.

The observed variation in diel transcriptional patterns between environmental protistan taxa suggested targeted allocation of transcriptional resources to different functional processes over the diel cycle. We sought to identify specific pathways with strong temporal partitioning by using Fisher’s Exact Test. We determined whether particular KEGG pathways were enriched in diel-oscillating gene families at each of the six time points by conducting tests on all unique taxa-time-pathway combinations. A total of 110,754 genus-time-pathway tests identified 78 significant taxa-time-pathway combination enrichments (BH < 0.05; Table 3 and Supplementary Data Sheet 5); these enrichments represent 28 total enriched KEGG pathways out of 430 pathways tested. These pathways encompass varied metabolic pathways including central carbon metabolism, lipid biosynthesis and degradation, protein biosynthesis and turnover, organellar processes, and signaling. We focused on KEGG pathways enriched in at least two of the nine representative environmental genera: “Photosynthesis,” “Carbon fixation in photosynthetic organisms,” “Porphyrin and chlorophyll metabolism,” “Proteasome,” “Protein processing in endoplasmic reticulum,” “TCA cycle,” “Circadian entrainment,” “Oxidative phosphorylation,” and “Ribosome” (Figure 4A).

**TABLE 3:** KEGG pathway enrichment analysis at the Genus level.

Class/*genus*	Peak (HST)	KEGG pathway
**Haptophyceae**		
*Prymnesium*	06:00	*Carbon fixation*, glycolysis/gluconeogenesis, *photosynthesis*, pentose phosphate pathway
	14:00	*Proteasome, protein processing in ER*, antigen processing and presentation
	18:00	*TCA cycle, oxidative phosphorylation*, thermogenesis
	22:00	Circadian entrainment
	02:00	*Ribosome*
*Phaeocystis*	06:00	*Photosynthesis, carbon fixation*
	14:00	*Protein processing in ER*
	18:00	*TCA cycle, proteasome*, thermogenesis
	02:00	Lysosome
**Dictyochophyceae**		
*Dictyocha*	06:00	*Photosynthesis, carbon fixation*, glycolysis/gluconeogenesis
	18:00	Thermogenesis
	22:00	*Ribosome*
*Florenciella*	–	*No enriched pathways at FDR* < *0.05*
*Pelagococcus*	18:00	Fatty acid degradation
	02:00	*Ribosome*
**Dinophyceae**		
*Karlodinium*	–	*No enriched pathways at FDR* < *0.05*
*Amoebophrya*	14:00	*TCA cycle, oxidative phosphorylation*
**Spirotrichea**		
*Strombidium*	06:00	*Ribosome*
	10:00	Biosynthesis of unsaturated fatty acids
	14:00	*Carbon fixation, TCA cycle*, glyoxylate and dicarboxylate metabolism
**Choanoflagellata**		
*Acanthoeca*	02:00	Amoebiasis

*Italics denote representative pathways plotted in Figure 4A. Human Disease pathways not shown.*

As expected, morning was characterized by enrichments in photosynthesis-related pathways. At dawn (06:00 HST), “Photosynthesis” and “Carbon fixation” pathways were enriched for diel oscillating gene families in phototrophs, including those with mixotrophic capabilities, with “Photosynthesis” the most frequently enriched pathway. A majority of gene families in the “Photosynthesis” pathway displayed peak transcript abundances at dawn (Figure 4A). At 10:00, “Porphyrin and chlorophyll metabolism” was the only pathway enriched in photosynthetic and mixotrophic lineages. The paucity of enrichments at 10:00 was consistent with the observed minimum in transcript peak across most environmental genera (Supplementary Figures 6, 7). One of the few whole pathway enrichments observed in dinoflagellate lineages was “Photosynthesis” in *Alexandrium* (Supplementary Data Sheet 5) at 10:00. Within the phagocytic taxa, *Strombidium* displayed significant enrichments in the “Ribosome” pathway at dawn (Figure 4A), similar to those seen in the sea squirt *Oikopleura* (Supplementary Data Sheet 5).

Later in the day, organisms shifted to protein processing and respiration-based pathways. The “Proteasome,” “Protein processing in endoplasmic reticulum,” and “TCA cycle” pathways were enriched at 14:00, with the latter pathway enriched in five haptophyte genera (Figure 4A and Supplementary Data Sheet 5). The haptophyte and ochrophyte genera had transcript abundance peaks in “Proteasome”-associated gene families in the afternoon (Figure 4A) whereas *Amoebophyra* and *Strombidium* displayed transcript abundance peaks in “TCA cycle”-associated gene families at this time point. By dusk (18:00), pathways involved in energy-yielding processes became transcriptionally prominent and included enrichments in “Oxidative phosphorylation” and additional enrichments in the TCA cycle. At this time the greatest number of pathway enrichments was detected, although the timing of transcript abundance peaks for individual gene families varied across genera. The ochrophytes displayed peaks in transcript abundance for individual TCA-associated gene families throughout the day, while the haptophytes also displayed peaks for TCA-associated genes before and at dawn (Figure 4A). “Oxidative phosphorylation” peak times also occurred across other time points in ochrophytes, but primarily between dusk to dawn in *Prymnesium* and *Phaeocystis*, afternoon in *Amoebophrya*, and before noon in *Strombidium* (Figure 4A).

The night timepoints (22:00 and 02:00) were characterized by enrichments in “Circadian entrainment,” “Oxidative phosphorylation,” and “Ribosome” pathways in most of the nine genera. *Pelagococcus* did not display enrichment in Circadian entrainment at any time point, was enriched in “Oxidative phosphorylation” during the day rather than at night and was enriched in the “Ribosome” pathway at the 02:00 time point.

Additional pathways were enriched in specific genera (Supplementary Data Sheet 5). The greatest number of pathway enrichments at the genus level was found in the haptophyte *Prymnesium* (16), followed by other haptophytes and ochrophyte genera (38 and 17 pathway-time enrichments in total, respectively). “Fatty acid biosynthesis” and “Fatty acid degradation” were enriched in a subset of the genus-level analyses. For example, biosynthesis of unsaturated fatty acids was enriched in the ciliate *Strombidium* at 10:00 (Supplementary Data Sheet 5), alluding to a build-up of energy storage reserves during the day. Enrichment of the “Thermogenesis” pathway in several genera (*Phaeocystis*, *Prymnesium*, and *Dictyocha*) was driven by peaks in transcripts encoding mitochondrial-targeted proteins. In addition, a subset of enriched pathways at this time point is characterized as Human Diseases in KEGG; these pathways contain ubiquitous gene families such as calmodulin, calcium channels, cytochrome oxidase, ATPase, and some components of the TCA cycle (Supplementary Data Sheet 5).

Five pathway enrichments were detected in Dinoflagellate genera with the most in *Amoebophrya* (“TCA cycle” and “Oxidative phosphorylation” were both enriched at 14:00); the other dinoflagellates had either one or no enrichments. The choanoflagellate genus *Acanthoeca* had few significant peak times in the most commonly enriched diel pathways (Figure 4A), even though nearly 16% of *Acanthoeca* gene families displayed diel oscillations in transcript abundances, a value comparable to *Prymnesium* (18%) (Figure 3A and Supplementary Data Sheets 3, 5). The only significant enrichment attributed to *Acanthoeca* was the disease pathway “Amoebiasis,” at 02:00.

The striking similarity in the overall patterns of the representative ochrophyte and haptophyte genera (Figure 4A) prompted an examination of transcription abundances for select gene families across the diel cycle, including those involved in “Photosynthesis” and “Chlorophyll metabolism,” the “TCA cycle,” “Ribosome,” and “Proteasome” pathways (Figure 4B). We focused on those gene families with transcript abundances that oscillated over the diel cycle and were detected in at least three genera from the representative ochrophytes (*Dictyocha*, *Florenciella*, and *Pelagococcus*) or haptophytes (*Phaeocystis* and *Prymnesium*). Each of the photosynthesis gene family transcript abundance patterns was remarkably consistent across genera. In general, transcript abundance was highest at dawn with a decline through the day culminating in a dusk minimum. Gene family transcripts in the TCA cycle pathway were also tightly correlated with an inverse transcriptional pattern to photosynthesis-related transcripts, with sharp peaks at the dusk time point and 02:00 or 06:00 minima. Two gene families involved in either protein synthesis (small subunit ribosomal protein S1, rpsA) or protein degradation (proteasome activator subunit 3, PSME3) had generally opposing phases, with rpsA transcripts at higher abundances in the dark until dusk and PSME3 transcripts with higher daytime abundances. These results suggested that the ochrophyte and haptophyte phytoplankton lineages maintained similar diel regulation of genes within these pathways.

To identify potentially distinguishing features of the major phytoplankton lineages, we further examined pathway enrichments at the class level corresponding to Haptophyceae, Dictyophyceae, Pelagophyceae, and Dinophyceae (Table 4). These four classes are inclusive of 6, 23, 1, and 3 of the 48 genera meeting cutoff criteria, respectively. A total of 45 KEGG pathway enrichments over the diel cycle were detected for Haptophyceae; Dictyophyceae and Pelagophyceae displayed both lower overall numbers of KOfams and sequence coverage with detection of 21 and 13 enrichments, respectively. Few diel enrichments were detected within Dinophyceae.

**TABLE 4:** KEGG pathway enrichment analysis at the class level.

Class	Peak (HST)	KEGG pathway
**Haptophyceae**		
	06:00	*Carbon fixation, photosynthesis*, fatty acid biosynthesis, glycolysis/gluconeogenesis, pentose phosphate pathway, p**henylalanine tyrosine and tryptophan biosynthesis**, **cysteine and methionine metabolism**, **alanine aspartate and glutamate metabolism, biotin metabolism, tropane piperidine and pyridine alkaloid biosynthesis**, **fructose and mannose metabolism**
	10:00	*Porphyrin and chlorophyll metabolism*
	14:00	*Protein processing in ER, proteasome*, **RNA transport**, spliceosome
	18:00	Spliceosome, **DNA replication,** *TCA cycle, oxidative phosphorylation***, cell cycle,** Synaptic vesicle cycle, **nucleotide excision repair**, *ribosome*, **meiosis**, fatty acid degradation, **mismatch repair,** thermogenesis, dopaminergic synapse
	22:00	**Calcium signaling pathway, cAMP signaling pathway**, thermogenesis, **Fc epsilon RI signaling pathway**, *circadian entrainment*, **natural killer cell mediated cytotoxicity**, oxytocin signaling pathway, **renin secretion**, **aldosterone synthesis and secretion**, **phospholipase D signaling pathway**, **MAPK signaling pathway**
	02:00	**Valine leucine and isoleucine biosynthesis**, **protein export**
**Dictyochophyceae**		
	06:00	*Carbon fixatio*n, glycolysis/gluconeogenesis, *photosynthesis*, **glycine serine and threonine metabolism**, pentose phosphate pathway, **sulfur metabolism**, **pyruvate metabolism**, **bacterial secretion system**
	10:00	*Porphyrin and chlorophyll metabolism, proteasome*, *photosynthesis*, **photosynthesis – antenna proteins**
	14:00	*Protein processing in ER*, **antigen processing and presentation**
	18:00	*TCA cycle*, **cardiac muscle contraction**, thermogenesis, *oxidative phosphorylation*
	22:00	*Ribosome*, **salivary secretion**, **NOD-like receptor signaling pathway**
	02:00	*No enriched pathways at FDR* < *0.05*
**Pelagophyceae**		
	06:00	*Carbon fixation*, *photosynthesis*, fatty acid biosynthesis,
	10:00	*Porphyrin and chlorophyll metabolism*
	14:00	*Protein processing in ER*
	18:00	*Ribosome*, *proteasome, TCA cycle*
	22:00	Dopaminergic synapse, **olfactory transduction**, oxytocin signaling pathway, **melanogenesis**, *circadian entrainment*
	02:00	*No enriched pathways at FDR* < *0.05*
**Dinophyceae**		
	06:00	*No enriched pathways at FDR* < *0.05*
	10:00	*No enriched pathways at FDR* < *0.05*
	14:00	**Drug metabolism – other enzymes**
	18:00	*Carbon fixation*, **lysosome**
	22:00	Synaptic vesicle cycle, **collecting duct acid secretion**, *oxidative phosphorylation, photosynthesis*
	02:00	*Carbon fixation*

*Bold, pathways specific to each class. Italics denote representative pathways plotted in Figure 4A. Human Disease pathways not shown. Pathways for each time per taxa are listed by decreasing significance order (FDR < 0.05).*

These analyses uncovered a number of distinguishing features of specific groups (Table 4). Specific enrichment of “Fructose and mannose metabolism” pathways in Haptophyceae may reflect an enhanced sensitivity for detecting enrichments in this well-covered class of organisms as this pathway is directly linked to glycolysis. The specific enrichment in “Pyruvate metabolism” in Dictyophyceae may reflect a similar routing of fixed carbon toward lipids. Haptophyceae was also specifically enriched at dawn in multiple amino acid metabolic pathways (“Alanine aspartate and glutamate metabolism,” “Phenylalanine, tyrosine and tryptophan biosynthesis,” and “Cysteine and methionine metabolism”) as well as “Biotin metabolism.” Other pathways with significant temporal enrichment only in Haptophyceae include “Aldosterone synthesis and secretion”; “Cellular senescence pathway”; “Valine, leucine, and isoleucine biosynthesis”; and “Protein export” pathways.

The Dictyophyceae specifically displayed dawn enrichments in both “Sulfur metabolism” and “Glycine, serine and threonine metabolism,” two pathways linked via the metabolite homoserine (Supplementary Figures 8, 9). Dictyophyceae also had a unique enrichment in the “Photosynthesis—antenna proteins” pathway. The morning-peaking gene families in the sulfur metabolism pathway form the majority of complete sulfate assimilation pathways in Dictyophyceae. Specific enrichment of “Antigen processing and presentation” and “Cardiac muscle contraction” pathways in the afternoon and at dusk, respectively, may reflect enrichments in general ATPase and transport functions. Enrichments identified only in Pelagophyceae were limited to “Olfactory transduction” and “Melanogenesis,” both at 22:00. Surprisingly, the Dinophyceae were enriched in “Carbon fixation in photosynthetic organisms” and “Photosynthesis” pathways during the night, whereas all other classes were enriched in these pathways in the early morning. Dinophyceae also specifically displayed enrichment in the “Lysosome” pathway at dusk (18:00), suggesting a dominant signal from heterotrophic or mixotrophic dinoflagellates, with nighttime partitioning of gene transcription likely associated with digestion of engulfed prey (Supplementary Figure 10). We note that aside from the “Drug metabolism” pathway, all enrichments identified in Dinophyceae are organelle localized (plastid or lysosome).

## Discussion

Microbial eukaryotes perform vital functions in the NPSG ecosystem, including phototrophy, heterotrophy, mixotrophy, and parasitism. The generation of time-, function-, and taxonomy-resolved environmental transcriptome bins in this study produced insights about community composition and abundance, the connection between transcriptome composition and trophic state, the degree of diel regulation utilized by these environmental taxa, and the timing of functional processes throughout the diel cycle. Because of the high amount of functional, temporal, and taxonomic resolution provided by the annotated metatranscriptomes, we have utilized an analytical hierarchy in this study: we begin with broad survey of all environmental bins, then narrow our global functional analysis to the most complete environmental genera, and further focus our examination of metabolism to abundant representative genera and prominent pathways. This includes the smaller sized genera (<7 μm diameter) that make significant contributions to daily productivity in the mixed layer of the NPSG (Freitas et al., 2020). Although there may be subtle variations in environmental factors contributing to the transcriptional differences between genera, our assumption is that diel cycles are the critical driver of microbial life in the NPSG, and we have constrained the majority of our statistical analysis to diel periodicity of gene families and the timing of pathways.

The environmental genera in this study can be roughly categorized as having either high, intermediate, or low levels of diel transcriptional regulation. The haptophyte *Phaeocystis* has the singular distinction of having the highest proportion of diel gene families. Although *Phaeocystis* was not the only pure phototroph captured by our study, it was the most abundant obligate phototroph among the complete genera bins, as such it is difficult to conclude from this study alone whether similarly high levels of diel regulation are a common phototroph strategy. Furthermore, though we chose to constrain our analysis to the genus-level or higher, there may be strain-level differences in diel regulation magnitude throughout the cosmopolitan *Phaeocystis*.

Organisms of comparable sequencing depth and completeness show similar levels of diel regulation in the “intermediate” range (∼12–22% of gene families being diel-oscillating) despite differences in their trophic state and evolutionary lineage. This includes non-dinoflagellate mixotrophic genera (including the haptophytes *Prymnesium* and *Chrysochromulina*, the dictyophyte *Florenciella*, and the non-constitutive mixotrophic ciliate *Strombidium*), the representative copepod genus *Lepeophtheirus*, and the choanoflagellate genus *Acanthoeca.* Diel transcriptome structuring in these genera may reflect physiological attunement to the diel cycle; in copepods, the high diel periodicity could be linked to diel vertical migration, but also reflects confounding issues from sub-group migration in and out of the mixed layer over diel cycles. The repeated observation of an “intermediate” level of diel regulation across disparate trophic modes and evolutionary lineages suggests that transcript synchronization to diel cycles is a common and advantageous strategy in the NPSG.

The “low” range of diel transcriptomes bin regulation (∼8% or less of gene families) is predominantly occupied by dinoflagellates, including mixotrophic and heterotrophic genera. The low degree of regulation in in dinoflagellates is consistent with studies showing a relatively dampened transcriptional response to environmental stimuli (Lin, 2011). Other strictly heterotrophic genera also had a low fraction of diel-regulated gene families, including the metazoan (animal) groups aside from *Lepeophtheirus* and heterotrophic protists other than *Acanthoeca*.

The coordination of protists to the diel period is also apparent in the distribution of diel transcript peak times. The highest proportions of transcript peak times occur at dusk, followed by dawn, underscoring the considerable metabolic re-arrangement of cells between light and dark periods. This has been observed previously in laboratory cultures: in the diatom *Thalassiosira pseudonana*, more gene transcript abundances peaked at dusk rather than dawn (Ashworth et al., 2013). The relative lack of gene family peak times at 10:00 HST, in particular, could be attributed to photo-protective purposes, minimizing transcription before the noon irradiation peak. This mid-morning minimum is also seen in prokaryotes: an earlier metatranscriptome study of the NPSG noted that the transcriptional minimum occurred closer to noon in contrast to MED4 culture transcriptomes (Ottesen et al., 2014). This “mid-day depression” could be a response to the detrimental effect of high UV radiation at mid-day in the surface layer and has been attributed to reduced growth, reduced DNA synthesis, and photochemical quenching in the picoplankton of the equatorial Pacific (Vaulot and Marie, 1999). The mid-day depression in transcription could also play a role in anti-viral defense, in that it restricts the replication of viral transcripts that are themselves tightly coordinated to diel cycles (Waldbauer et al., 2012).

Functional analysis of diel-regulated gene families allowed us to infer how protist lineages allocate transcriptional resources to functional processes over the diel cycle. Some metabolic features of phototrophs appear to be common strategies, such as dawn peaks in photosynthesis and energy storage pathways, and up-regulation of DNA synthesis and cell cycle elements at dusk. This strategy has been observed in diatoms and haptophytes previously. In the diatom *T. pseudonana*, genes encoding cell division, DNA replication and repair, carbon metabolism, and oxidative phosphorylation enzymes are highly expressed at dusk, while at dawn transcripts involved in Photosynthesis, Carbon fixation, and Ribosomes were higher (Ashworth et al., 2013); this general phototrophic strategy was also observed in metatranscripts from the calcifying haptophyte *E. huxleyi* (Hernández et al., 2020). Transcriptional evidence for the cycling of carbon is consistent with *in situ* field measurements of triacylglycerol in the NPSG that show increasing concentrations in cellular biomass through the day and decreases after nightfall (Becker et al., 2018). In particular, the striking similarity in transcriptional patterns between haptophytes and ochrophytes, both members of the Chromealveolate supergroup that separated an estimated 1 billion years ago (Yoon et al., 2004), implies selective pressure to conserve diel regulation of key cellular processes.

Taxa-specific metabolic features may be important in understanding the fate of carbon in the surface layer as a function of community composition. At the class-level, the haptophytes (Haptophyceae) constitute a “best case scenario” for diel pathway enrichments, as a combination of high diel regulation and population abundance contribute to sufficiently deep sequencing of haptophyte transcripts. The coordination of protein biosynthesis and turnover pathways to the diel cycle in haptophytes highlights the importance of these processes in diel cycles. The focused peaks of these pathway peaks in the afternoon suggests a large-scale proteomic remodeling prior to the evening metabolic switch, possibly involving the tagging and degradation of photosynthesis-related proteins in the dark hours when they are no longer useful. Intensive recycling of the proteome in the N-limited gyre could be an advantageous strategy to alleviate nitrogen stress, allowing nitrogen to be recycled between alternating proteomic regimes in a manner similar to the “hot-bunking” of iron atoms between anti-phase metalloenzymes in *Crocosphaera* (Saito et al., 2011). The functional pathway enrichments specific to dictyophytes, such as “Sulfur metabolism”; “Pyruvate metabolism”; and “Glycine, serine, and threonine metabolism” are evidence of additional lineage-specific temporal partitioning of metabolic processes. The “Sulfur metabolism” pathway includes sulfate assimilation, which is dominated by morning transcriptional peaks in this group. The connection of the “Sulfur metabolism” pathway to “Glycine, serine, and threonine Metabolism” through the metabolite homoserine highlights important connections between diel-enriched metabolic features. Many protists participate in diel cycling of sulfonated compounds (Durham et al., 2019), and these results hint at a possibly unexplored role of dictyophytes in sulfur cycling in the oceans. Future studies into the apparent metabolic differences between major protist lineages would benefit from metabolite- or protein-level data and fine-scale targeted investigation of specific metabolic pathways.

This study lends new perspective into dinoflagellate genetic regulation, which has significantly diverged from the transcriptional-level control utilized by most other eukaryotic lineages. The low proportion of diel gene families in dinoflagellate taxonomic bins is consistent with other studies showing a loss of transcriptional regulation in dinoflagellates (Kojima et al., 2011; Lin, 2011). The presence of 5′ spliced leaders, a transcript modification observed extensively in all major dinoflagellate orders, is a dinoflagellate adaptation that has been invoked as an alternative mechanism of gene regulation (Zhang et al., 2007). As expected from their low level of diel regulation, the dinoflagellates did not return many significant pathway enrichments at any phylogenetic level of investigation. The few enriched dinoflagellate pathways are intriguing, as they contrast the generally minimum transcriptional regulation in dinoflagellates. At the class level, we observed organelle-targeted enrichments in three pathways (“Photosynthesis,” “Carbon fixation,” and “Lysosome”), and it would be interesting to investigate whether the gene families involved use 5′ spliced leaders in a similar manner as cytosolic transcripts. Along with the enrichments, we found the timing of the “Photosynthesis” and “Carbon fixation” pathways (evening) to be surprising, as all other photosynthetic groups maintained these pathways with predominantly morning (06:00) peaks. Proteomic studies have been helpful in describing the altered function of environmental dinoflagellates across spatial gradients (Cohen et al., 2021) and future proteomic studies of dinoflagellates with diel temporal resolution would be useful in determining the translational offset of dinoflagellate proteins from their transcript peaks over these cycles. Within the dinoflagellates, the notable outlier was Syndiniales (represented here by genus *Amoebophyra*), an order of obligate parasitoids that infect dinoflagellates and other marine organisms (Guillou et al., 2008). This lineage branched off from other dinoflagellates early in their evolution, before the transcriptional adaptations that characterize more-derived dinoflagellates. Conservation of diel periodicity in *Amoebophyra* may provide hints into the ancestral transcriptional regulation of dinoflagellates that appear to have lost the course of their evolutionary history in most other genera.

The proportion of taxonomy and function-calling for the assembled contigs in our dataset is comparable to the Tara Oceans Initiative eukaryotic gene catalog, which assigned about 25% of putative coding frames a functional annotation (Pfam) and about ∼50% of the contigs a taxonomy at any level (Carradec et al., 2018). Despite some differences in our reference databases and separate functional databases, we see nearly identical annotation results in our data, with 54 and 25.7% of contigs receiving confident taxonomical and functional (KEGG Orthology) annotations, respectively. Much remains to be discovered in the “microbial dark matter” of sequence data that has no match to extant taxonomic or functional databases. As novel organisms continue to be sequenced and functional annotation databases are expanded in the future, the annotation of the raw transcript data generated by this study can continue to be improved and exploited to gain further scientific insight. Regardless, the thousands of currently catalogd gene family profiles provide us rich detail into the presence and timing of known metabolic pathways.

This study reveals the diel transcriptional dynamics of eukaryotic protists in the surface layers of the NPSG, elucidating common patterns and striking differences in the transcriptional phenotypes of the most abundant protists in the surface community. Taking all of these results in aggregate, a picture emerges of a eukaryotic community tightly orchestrated to the daily rhythms of sunlight, with phototrophic organisms structuring their transcriptomes around the clock to harness and store solar energy during the day, and to replicate, divide, and possibly exchange signaling molecules at night. Mixotrophs are abundant and vital members of the protist community, and employ most of the core metabolic strategies as phototrophs. Understanding the most prevalent metabolic strategies employed by microbial eukaryotes in conjunction with the differences that distinguish them is critical to furthering our understanding of how carbon, nutrients, and energy flow through the surface ocean ecosystem.

## Data Availability Statement

KM1513 cruise information, plots, and associated environmental data for the HOE Legacy II cruise can be found online at http://hahana.soest.hawaii.edu/hoelegacy/hoelegacy.html. Raw metatranscriptome short-read sequence data is available in the NCBI Sequence Read Archive under BioProject ID PRJNA492142. Assembled contigs are deposited in Zenodo (https://doi.org/10.5281/zenodo.5009803). Code associated with this project is available on Github (https://github.com/armbrustlab/diel_eukaryotes).

## Author Contributions

RG, SC, BD, and EA conceived and designed the research project. RG and SC conducted the metatranscriptomic analyses. All authors contributed to data interpretation and the writing of the manuscript.

## Conflict of Interest

The authors declare that the research was conducted in the absence of any commercial or financial relationships that could be construed as a potential conflict of interest.

## Publisher’s Note

All claims expressed in this article are solely those of the authors and do not necessarily represent those of their affiliated organizations, or those of the publisher, the editors and the reviewers. Any product that may be evaluated in this article, or claim that may be made by its manufacturer, is not guaranteed or endorsed by the publisher.

## Footnotes

1
http://roseobase.org/data/

## Supplementary Material

Supplementary Figure 1 Sequence length distribution of ∼25 million quality-controlled Trinity-assembled contigs. Contigs less than 1,000 nucleotides in length are shown; approximately 482,000 contigs (1.9% of total) are longer than 1,000 nucleotides and range from 1,001 to 18,833 nucleotides.

Supplementary Figure 2 Taxonomic assignments by primary Linnean ranks. Assignments totaling less than 2.5% of total placements for each rank are aggregated into “Other.” Not all taxa have higher-level rank assignments in the NCBI taxonomy; phylum and class rank assignments do not exist for many lineages and were not manually assigned.

Supplementary Figure 3 Frequency of unique KEGG ontologies (KOs) in eukaryotic MarineRefII reference taxa. *x*-axis: number of unique KOs found in each taxa after mapping to KEGG’s KOfam library of HMMer profiles. *y*-axis: frequency of occurrence. Dashed vertical line: minimum threshold of 900 KOs required for determination of “core KOs” present in >95% of reference taxa.

Supplementary Figure 4 *E*-value distributions for DIAMOND taxonomy assignments for environmental genera bins. Counts include assignments directly at the genus level and lower nodes (e.g., species under a genus).

Supplementary Figure 5 Non-metric multidimensional scaling (NMDS) ordination of Bray-Curtis from row-normalized KOfam counts. NMDS ordination performed independently on gene families belonging to each of 48 environmental genera that met completeness criteria. Mean stress of 48 NMDS = 0.127 ± 0.028 stdev.

Supplementary Figure 6 Gene family transcript peak times for 48 protist genera across the diel cycle. Gene families are significantly periodic with an FDR < 0.05. *x*-axis: clock hour in 24-h day. *y*-axis: percentage of significantly diel KOs with a peak at this time.

Supplementary Figure 7 Normalized abundance heat map of significantly periodic gene families from 48 protist genera. Yellow and gray bars denote light (06:00, 10:00, and 14:00 HST) and dark (18:00, 22:00, and 02:00 HST) periods, respectively. Each row corresponds to a gene family, ordered by hierarchical clustering of abundance patterns. Color corresponds to row-normalized abundance values for each gene family. Colored dot by genus name corresponds to Lineage from Figure 3A. The order and presence of gene families is not maintained between facets.

Supplementary Figure 8 Peak times for sulfur metabolism transcripts in dictyophytes. Most enzymes in this pathway peak at dawn, including the sulfate to sulfite component of assimilatory sulfate reduction. Metabolic maps were generated using KEGG Color Mapper (https://www.kegg.jp/kegg/mapper/color.html). Enzymes with significant periodicity are colored according to their peak time as determined through RAIN analysis of transcript abundance. Warm colors indicate peaks in dawn/daylight hours: yellow, 06:00 HST; orange, 10:00; red, 14:00. Cool colors indicate peaks in dusk/night hours: purple, 18:00 HST; blue, 22:00; green, 02:00.

Supplementary Figure 9 Peak times for glycine, serine, and threonine metabolism transcripts in dictyophytes. Dictyophytes maintain peak times for most of the enzymes in this pathway throughout daylight hours. Metabolic maps were generated using KEGG Color Mapper (https://www.kegg.jp/kegg/mapper/color.html). Enzymes with significant periodicity are colored according to their peak time as determined through RAIN analysis of transcript abundance. Warm colors indicate peaks in dawn/daylight hours: yellow, 06:00 HST; orange, 10:00; red, 14:00. Cool colors indicate peaks in dusk/night hours: purple, 18:00 HST; blue, 22:00; green, 02:00.

Supplementary Figure 10 Peak times for lysosome transcripts in dinoflagellates. Most periodic lysosomal enzymes have peaks in the afternoon (14:00), dusk (18:00), and early evening (22:00). Metabolic maps were generated using KEGG Color Mapper (https://www.kegg.jp/kegg/mapper/color.html). Enzymes with significant periodicity are colored according to their peak time as determined through RAIN analysis of transcript abundance. Warm colors indicate peaks in dawn/daylight hours: yellow, 06:00 HST; orange, 10:00; red, 14:00. Cool colors indicate peaks in dusk/night hours: purple, 18:00 HST; blue, 22:00; green, 02:00.
