# Supplementary figures and images for: Diel-Regulated Transcriptional Cascades of Microbial Eukaryotes in the North Pacific Subtropical Gyre
Source: Front Microbiol. 2021 Sep 29;12:682651. doi: 10.3389/fmicb.2021.682651 (PMC8511712; doi:10.3389/fmicb.2021.682651)

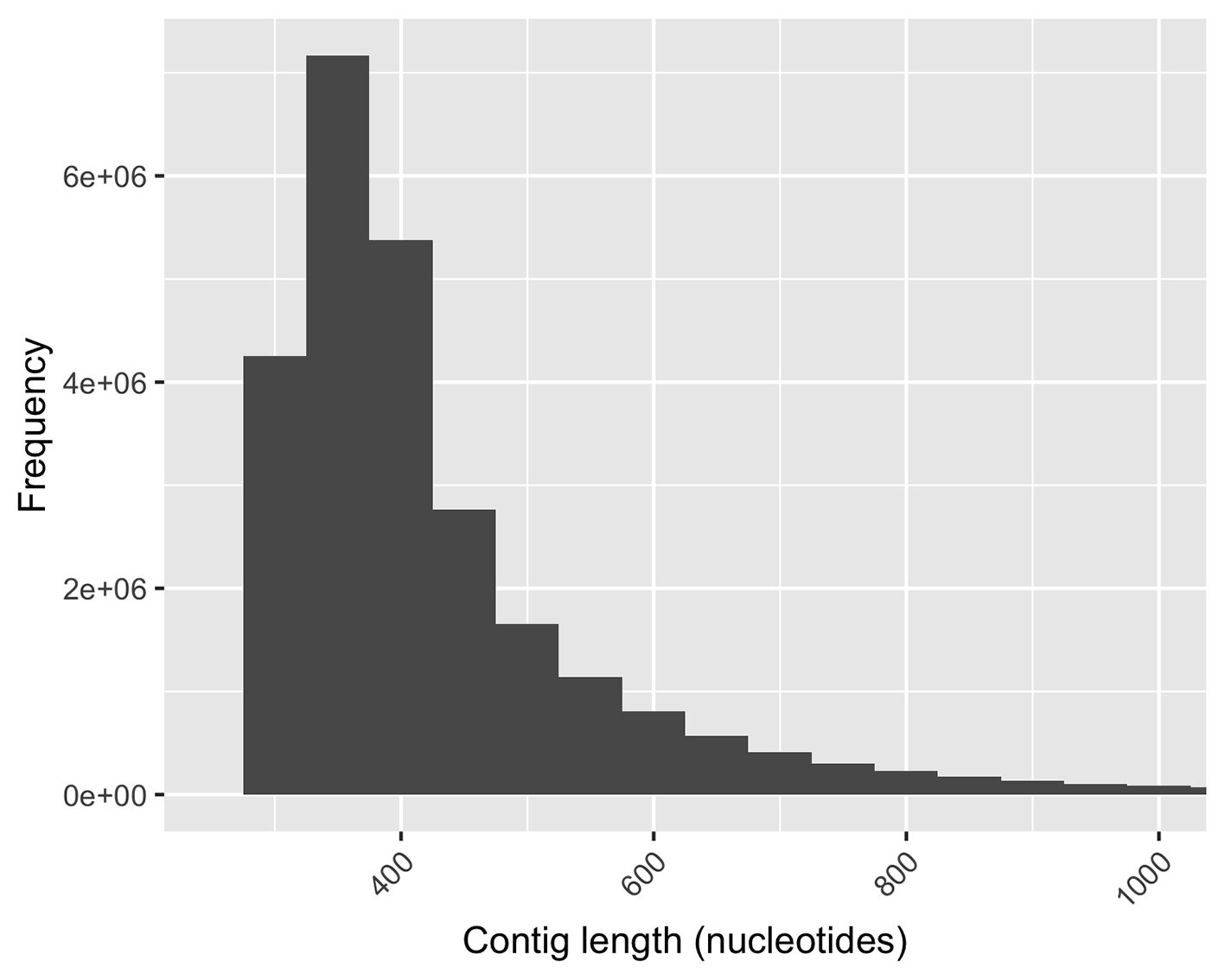

Supplement: Supplementary Figure 1 — Sequence length distribution of ∼25 million quality-controlled Trinity-assembled contigs. Contigs less than 1,000 nucleotides in length are shown; approximately 482,000 contigs (1.9% of total) are longer than 1,000 nucleotides and range from 1,001 to 18,833 nucleotides. [file Image_1.TIFF]

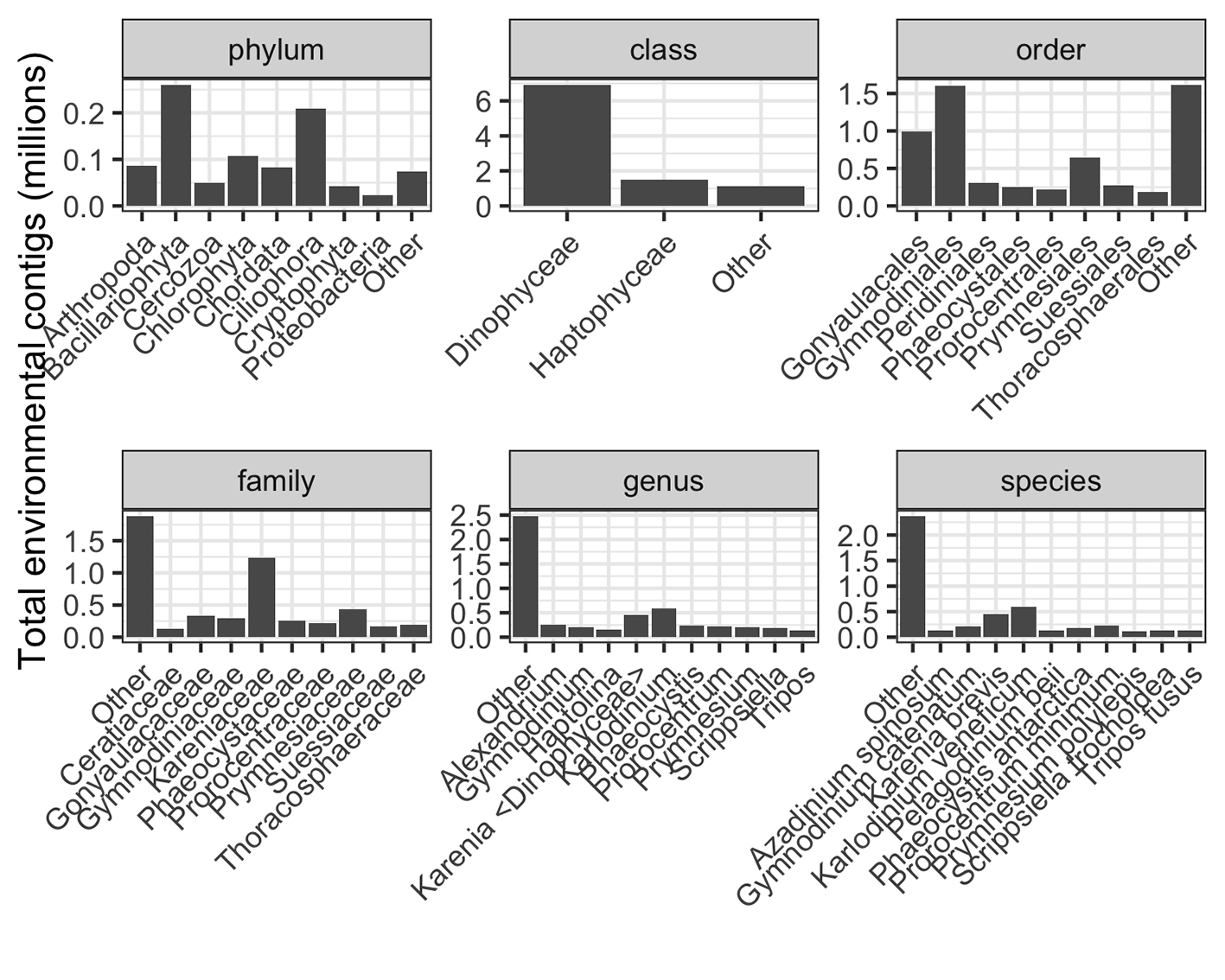

Supplement: Supplementary Figure 2 — Taxonomic assignments by primary Linnean ranks. Assignments totaling less than 2.5% of total placements for each rank are aggregated into “Other.” Not all taxa have higher-level rank assignments in the NCBI taxonomy; phylum and class rank assignments do not exist for many lineages and were not manually assigned. [file Image_2.TIFF]

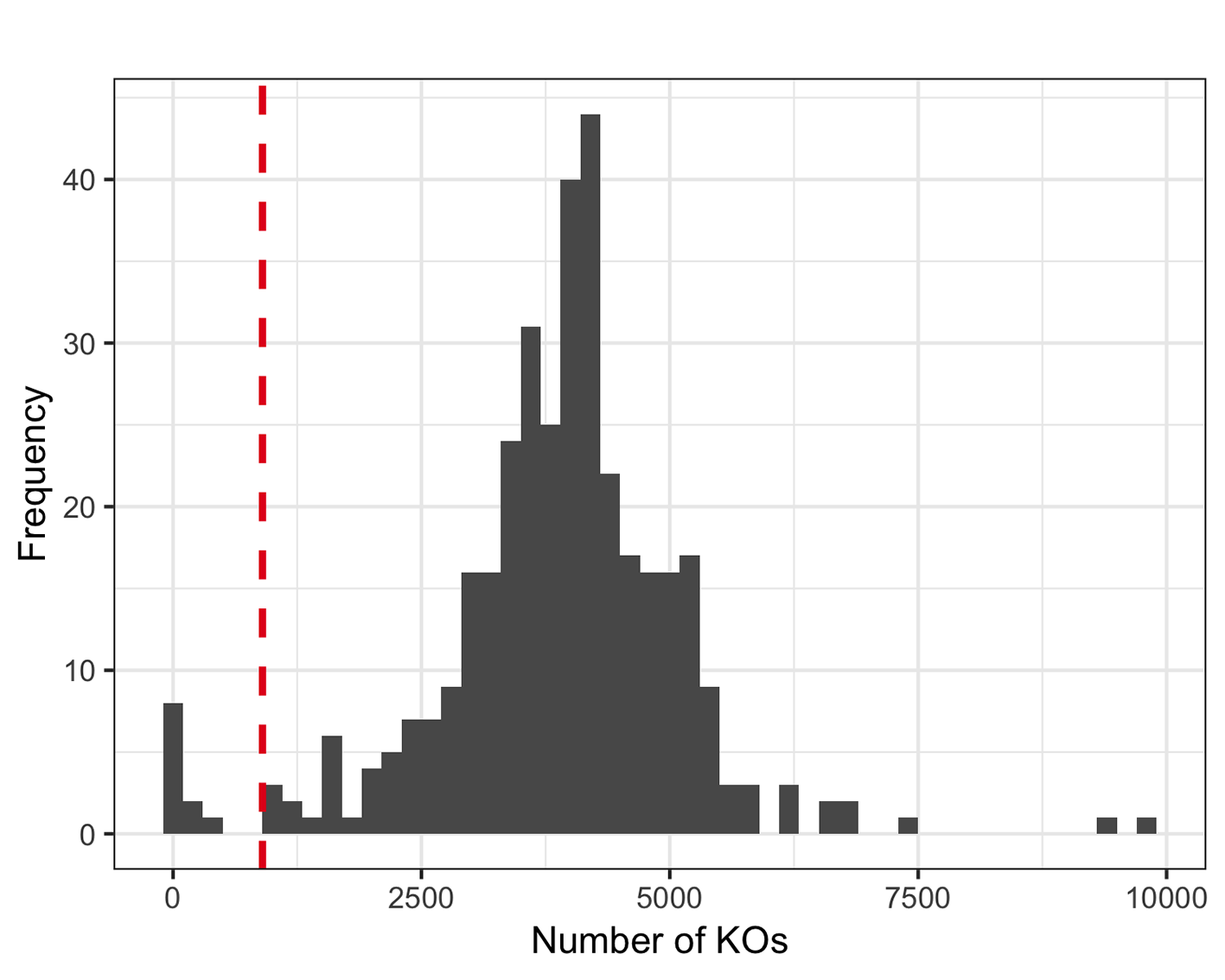

Supplement: Supplementary Figure 3 — Frequency of unique KEGG ontologies (KOs) in eukaryotic MarineRefII reference taxa. x-axis: number of unique KOs found in each taxa after mapping to KEGG’s KOfam library of HMMer profiles. y-axis: frequency of occurrence. Dashed vertical line: minimum threshold of 900 KOs required for determination of “core KOs” present in >95% of reference taxa. [file Image_3.TIFF]

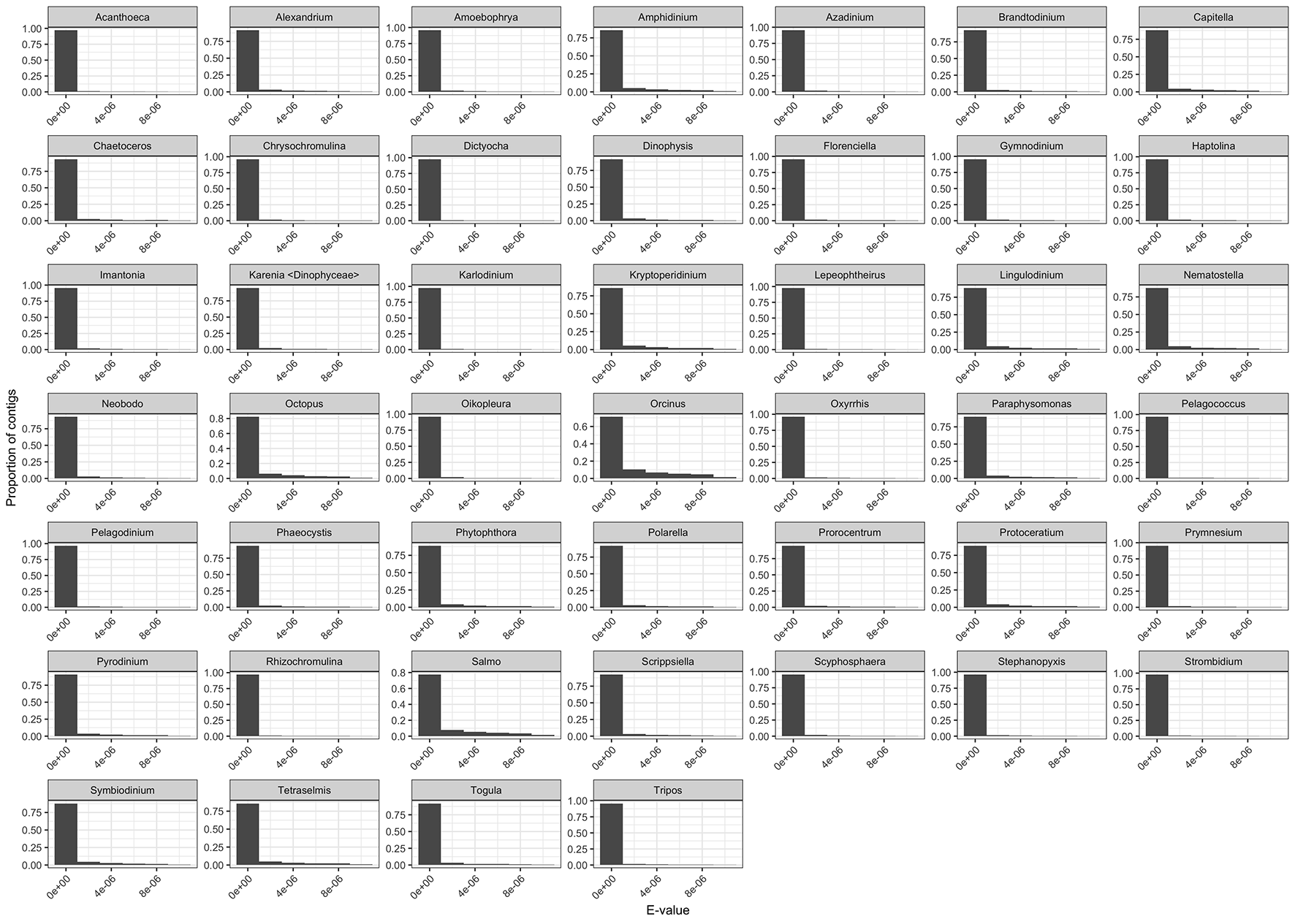

Supplement: Supplementary Figure 4 — E-value distributions for DIAMOND taxonomy assignments for environmental genera bins. Counts include assignments directly at the genus level and lower nodes (e.g., species under a genus). [file Image_4.TIFF]

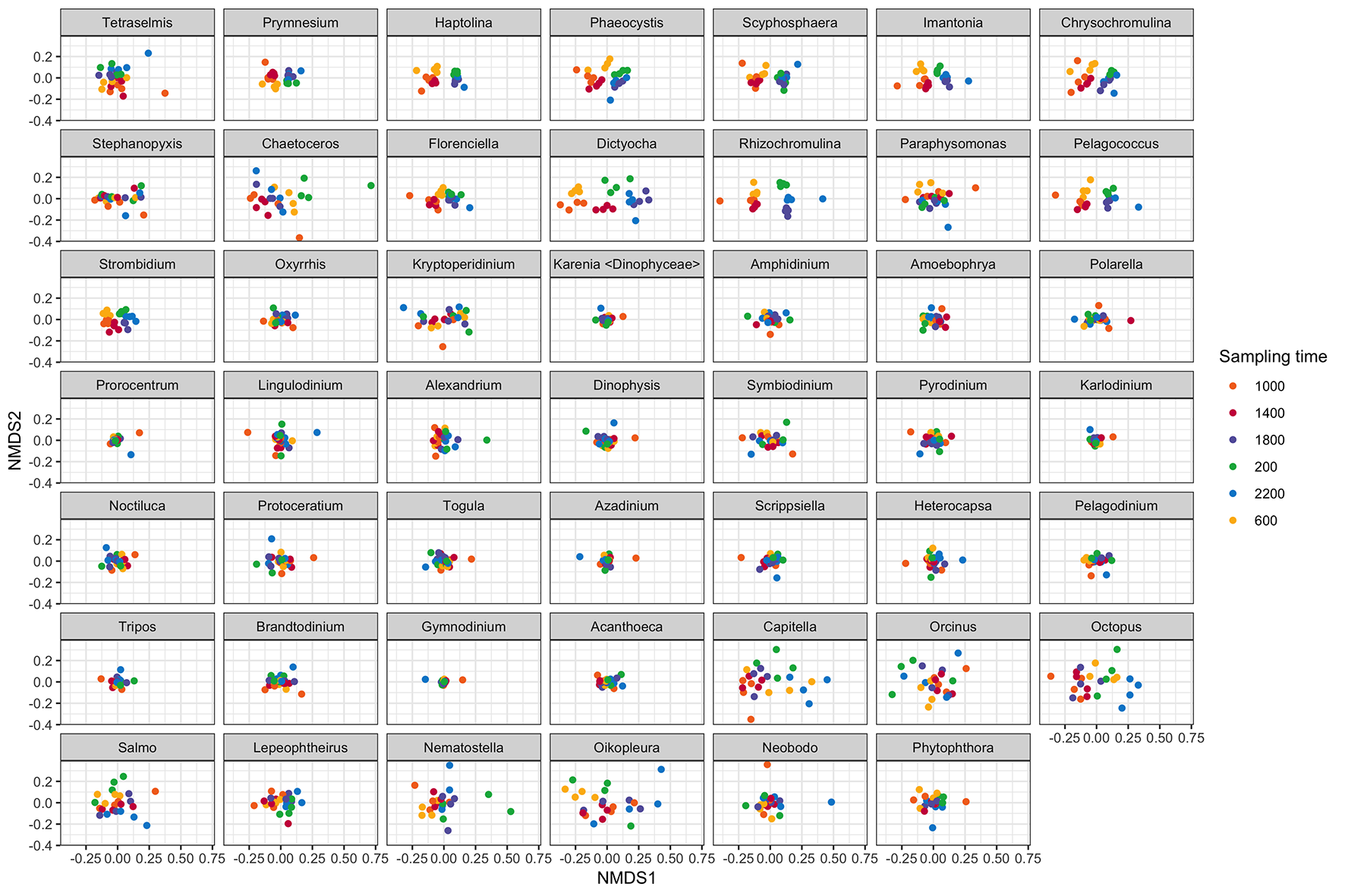

Supplement: Supplementary Figure 5 — Non-metric multidimensional scaling (NMDS) ordination of Bray-Curtis from row-normalized KOfam counts. NMDS ordination performed independently on gene families belonging to each of 48 environmental genera that met completeness criteria. Mean stress of 48 NMDS = 0.127 ± 0.028 stdev. [file Image_5.TIFF]

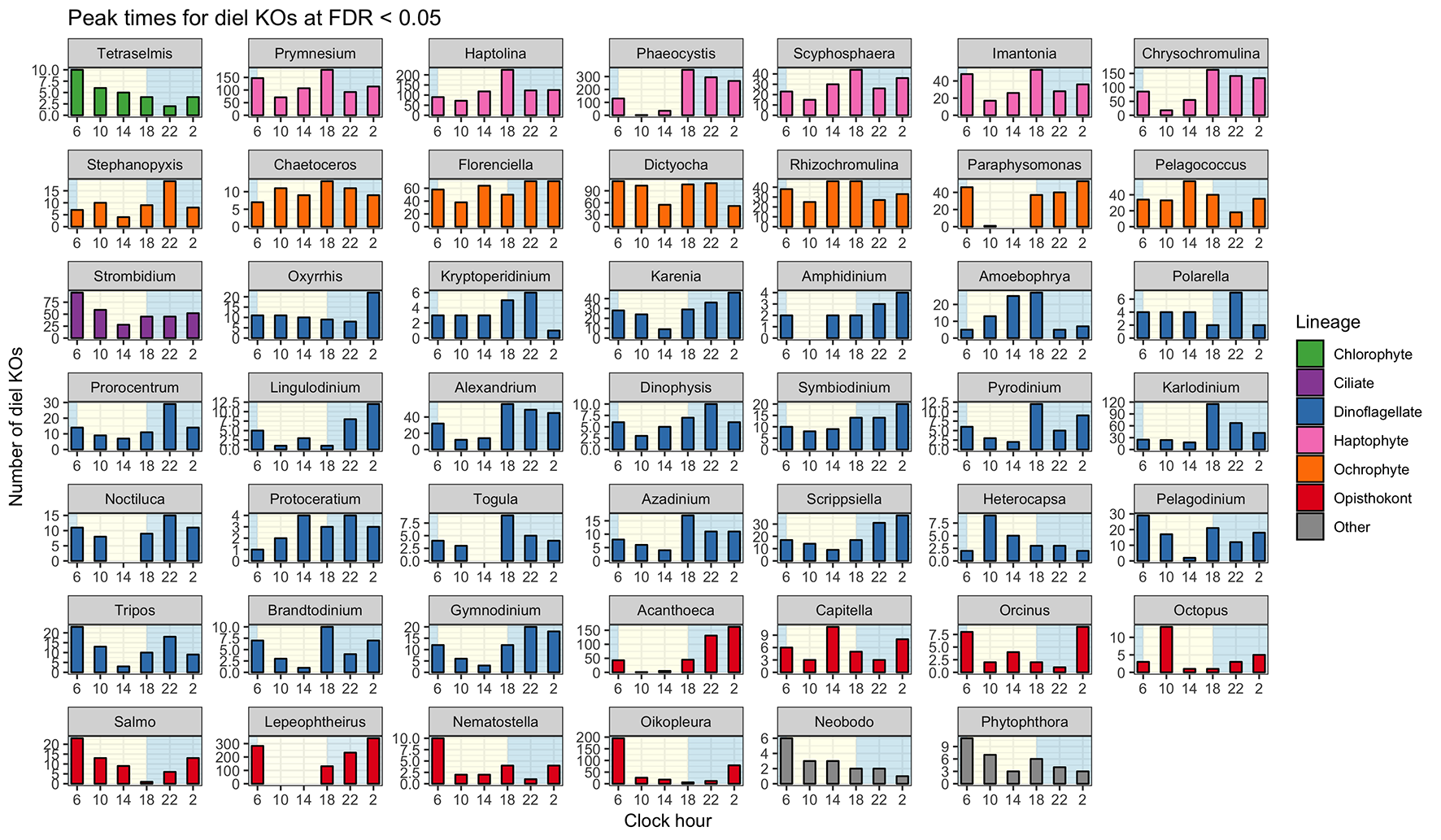

Supplement: Supplementary Figure 6 — Gene family transcript peak times for 48 protist genera across the diel cycle. Gene families are significantly periodic with an FDR < 0.05. x-axis: clock hour in 24-h day. y-axis: percentage of significantly diel KOs with a peak at this time. [file Image_6.TIFF]

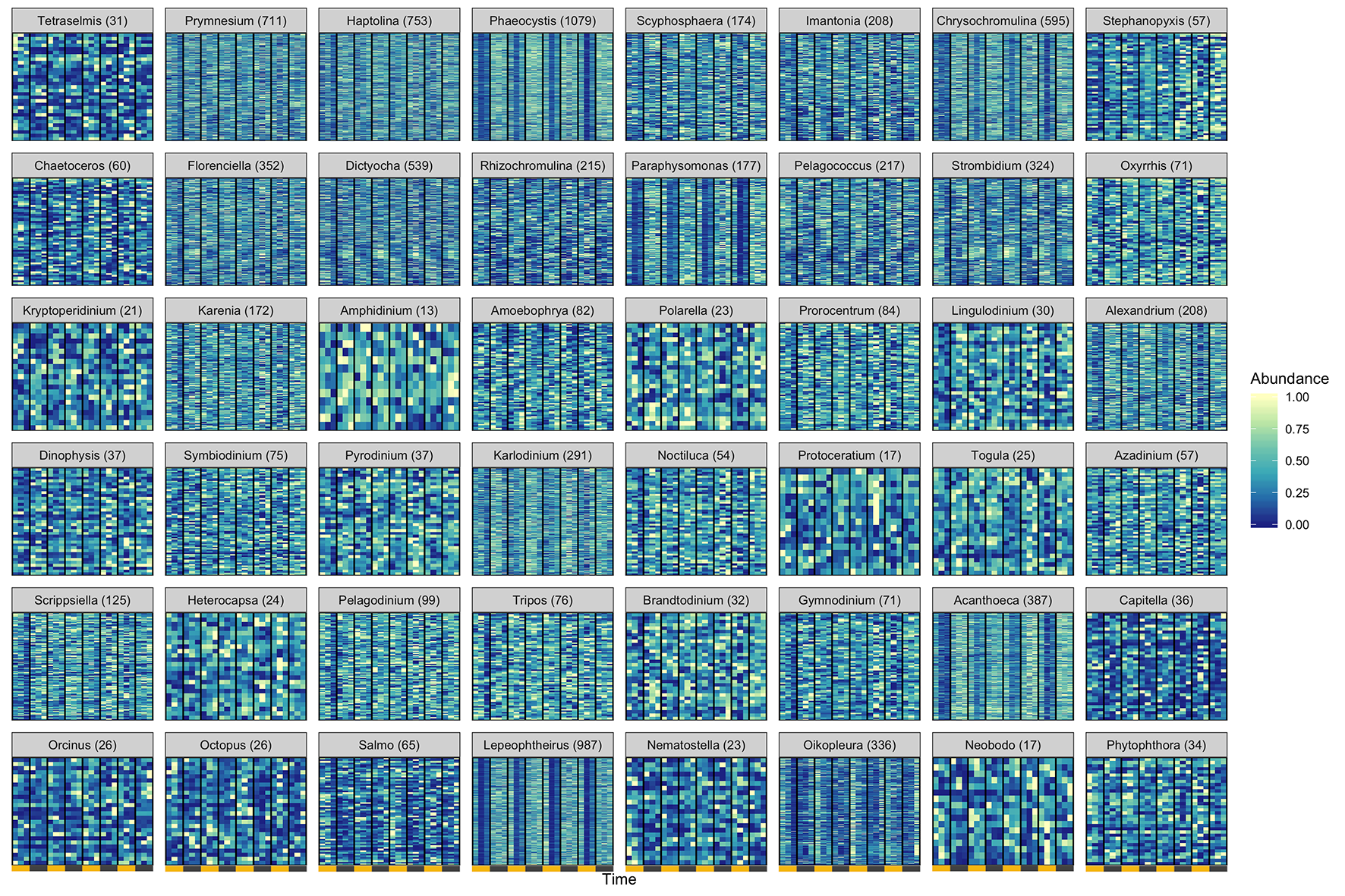

Supplement: Supplementary Figure 7 — Normalized abundance heat map of significantly periodic gene families from 48 protist genera. Yellow and gray bars denote light (06:00, 10:00, and 14:00 HST) and dark (18:00, 22:00, and 02:00 HST) periods, respectively. Each row corresponds to a gene family, ordered by hierarchical clustering of abundance patterns. Color corresponds to row-normalized abundance values for each gene family. Colored dot by genus name corresponds to Lineage from Figure 3A. The order and presence of gene families is not maintained between facets. [file Image_7.TIFF]

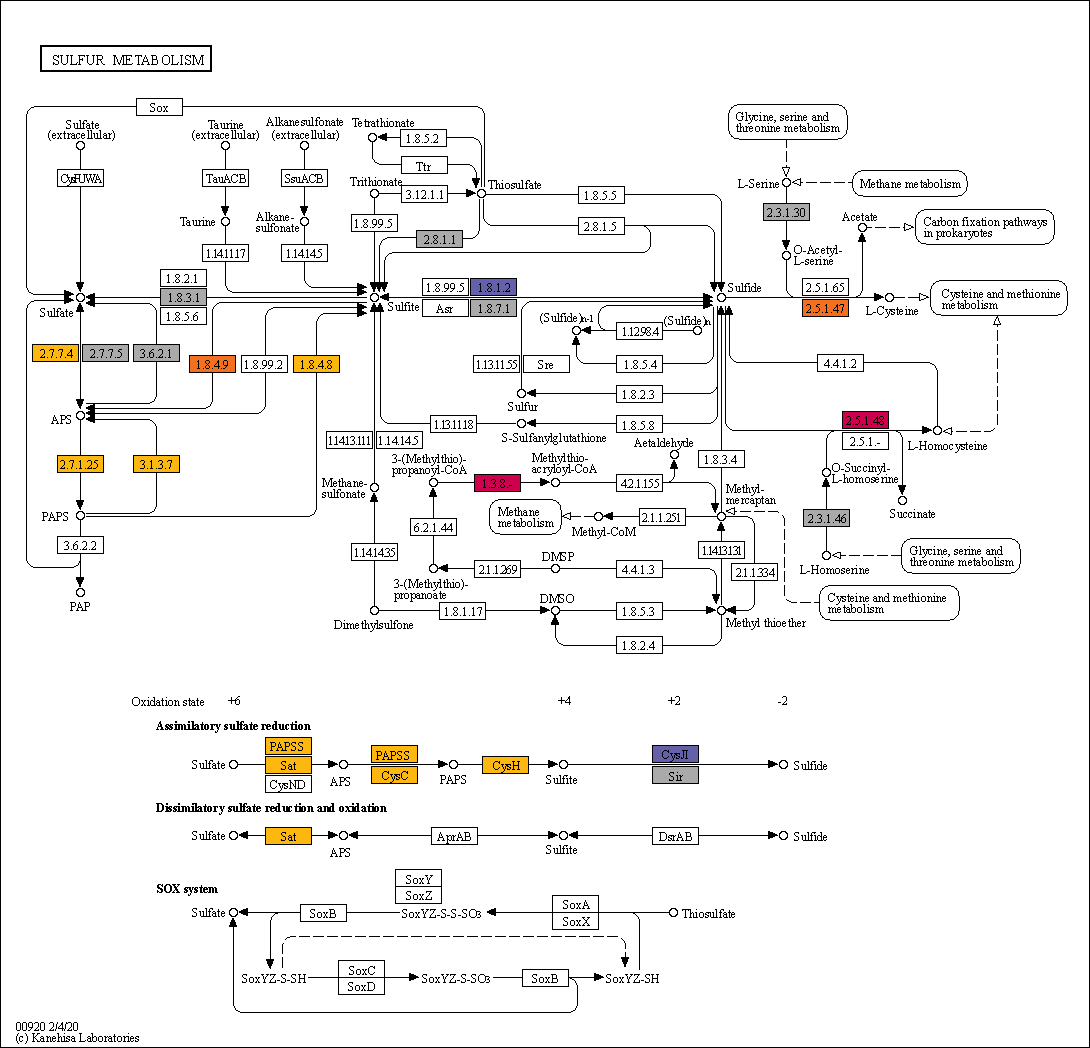

Supplement: Supplementary Figure 8 — Peak times for sulfur metabolism transcripts in dictyophytes. Most enzymes in this pathway peak at dawn, including the sulfate to sulfite component of assimilatory sulfate reduction. Metabolic maps were generated using KEGG Color Mapper (https://www.kegg.jp/kegg/mapper/color.html). Enzymes with significant periodicity are colored according to their peak time as determined through RAIN analysis of transcript abundance. Warm colors indicate peaks in dawn/daylight hours: yellow, 06:00 HST; orange, 10:00; red, 14:00. Cool colors indicate peaks in dusk/night hours: purple, 18:00 HST; blue, 22:00; green, 02:00. [file Image_8.TIFF]

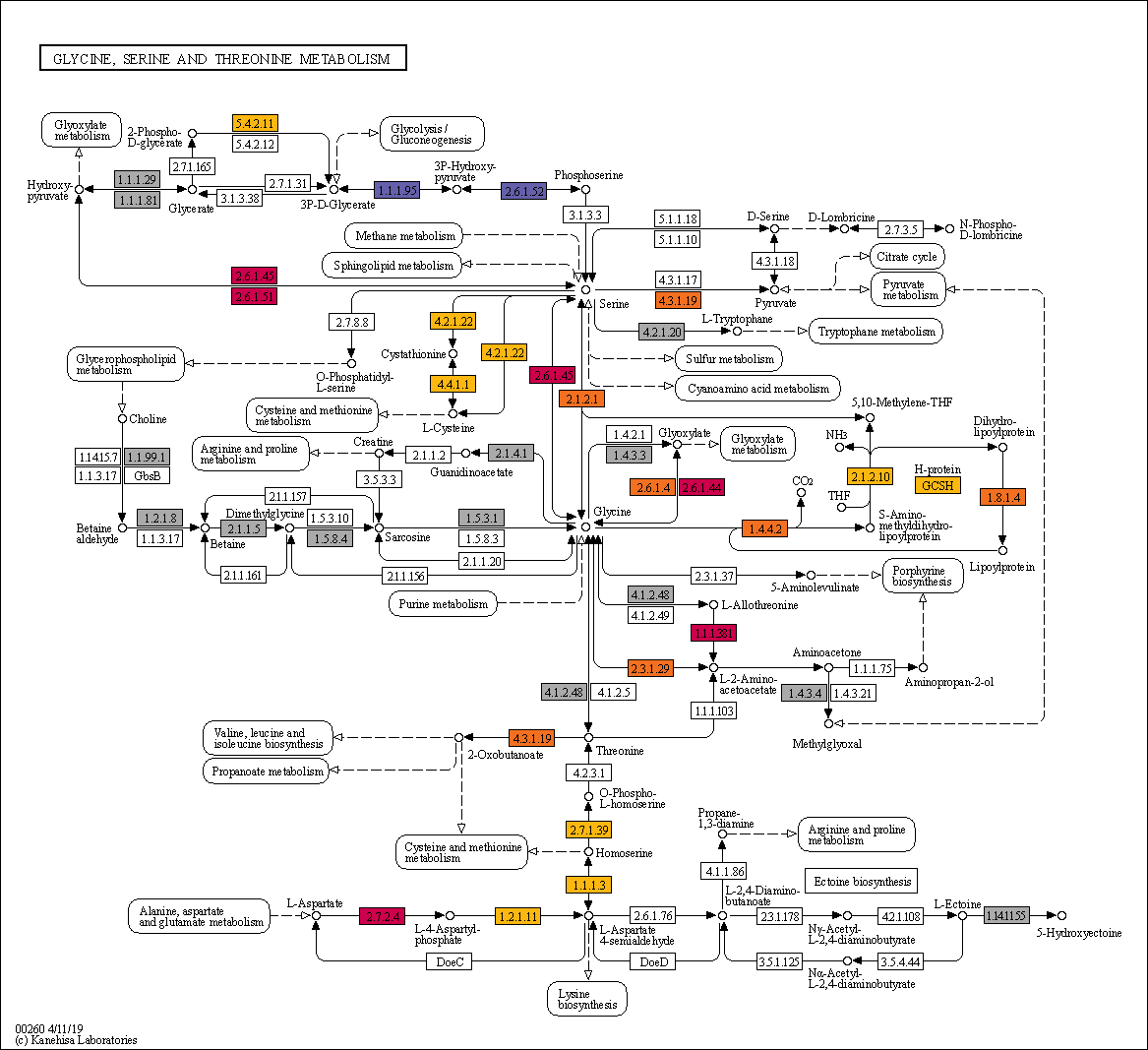

Supplement: Supplementary Figure 9 — Peak times for glycine, serine, and threonine metabolism transcripts in dictyophytes. Dictyophytes maintain peak times for most of the enzymes in this pathway throughout daylight hours. Metabolic maps were generated using KEGG Color Mapper (https://www.kegg.jp/kegg/mapper/color.html). Enzymes with significant periodicity are colored according to their peak time as determined through RAIN analysis of transcript abundance. Warm colors indicate peaks in dawn/daylight hours: yellow, 06:00 HST; orange, 10:00; red, 14:00. Cool colors indicate peaks in dusk/night hours: purple, 18:00 HST; blue, 22:00; green, 02:00. [file Image_9.TIFF]

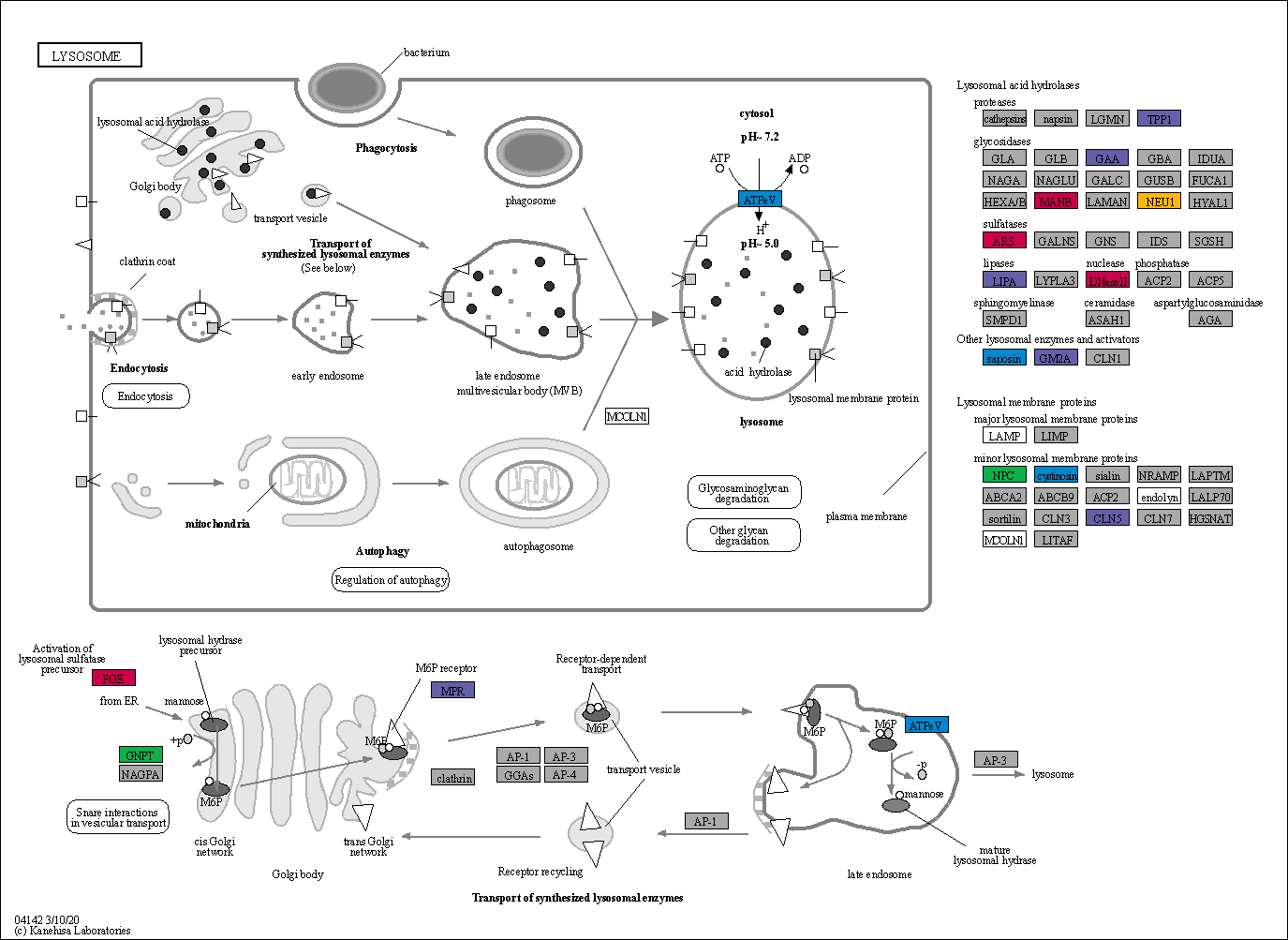

Supplement: Supplementary Figure 10 — Peak times for lysosome transcripts in dinoflagellates. Most periodic lysosomal enzymes have peaks in the afternoon (14:00), dusk (18:00), and early evening (22:00). Metabolic maps were generated using KEGG Color Mapper (https://www.kegg.jp/kegg/mapper/color.html). Enzymes with significant periodicity are colored according to their peak time as determined through RAIN analysis of transcript abundance. Warm colors indicate peaks in dawn/daylight hours: yellow, 06:00 HST; orange, 10:00; red, 14:00. Cool colors indicate peaks in dusk/night hours: purple, 18:00 HST; blue, 22:00; green, 02:00. [file Image_10.TIFF]
